# Molecular basis for the recognition of 24-(*S*)-hydroxycholesterol by integrin αvβ3

**DOI:** 10.1038/s41598-023-36040-4

**Published:** 2023-06-06

**Authors:** Jeevan B. Gc, Justin Chen, Swechha M. Pokharel, Indira Mohanty, Charles Mariasoosai, Peter Obi, Paul Panipinto, Smarajit Bandyopadhyay, Santanu Bose, Senthil Natesan

**Affiliations:** 1 Department of Pharmaceutical Sciences, College of Pharmacy and Pharmaceutical Sciences, Washington State University, Spokane, WA 992020 USA; 2 Department of Veterinary Microbiology and Pathology, College of Veterinary Medicine, Washington State University, Pullman, WA 99210 USA; 3 Molecular Biotechnology Core Laboratory, Lerner Research Institute, Cleveland Clinic, Cleveland, OH 44195 USA

**Keywords:** Biophysics, Immunology

## Abstract

A growing body of evidence suggests that oxysterols such as 25-hydroxycholesterol (25HC) are biologically active and involved in many physiological and pathological processes. Our previous study demonstrated that 25HC induces an innate immune response during viral infections by activating the integrin-focal adhesion kinase (FAK) pathway. 25HC produced the proinflammatory response by binding directly to integrins at a novel binding site (site II) and triggering the production of proinflammatory mediators such as tumor necrosis factor-α (TNF) and interleukin-6 (IL-6). 24-(S)-hydroxycholesterol (24HC), a structural isomer of 25HC, plays a critical role in cholesterol homeostasis in the human brain and is implicated in multiple inflammatory conditions, including Alzheimer’s disease. However, whether 24HC can induce a proinflammatory response like 25HC in non-neuronal cells has not been studied and remains unknown. The aim of this study was to examine whether 24HC produces such an immune response using in silico and in vitro experiments. Our results indicate that despite being a structural isomer of 25HC, 24HC binds at site II in a distinct binding mode, engages in varied residue interactions, and produces significant conformational changes in the specificity-determining loop (SDL). In addition, our surface plasmon resonance (SPR) study reveals that 24HC could directly bind to integrin αvβ3, with a binding affinity three-fold lower than 25HC. Furthermore, our in vitro studies with macrophages support the involvement of FAK and NFκB signaling pathways in triggering 24HC-mediated production of TNF. Thus, we have identified 24HC as another oxysterol that binds to integrin αvβ3 and promotes a proinflammatory response via the integrin-FAK-NFκB pathway.

## Introduction

**Figure 1:**
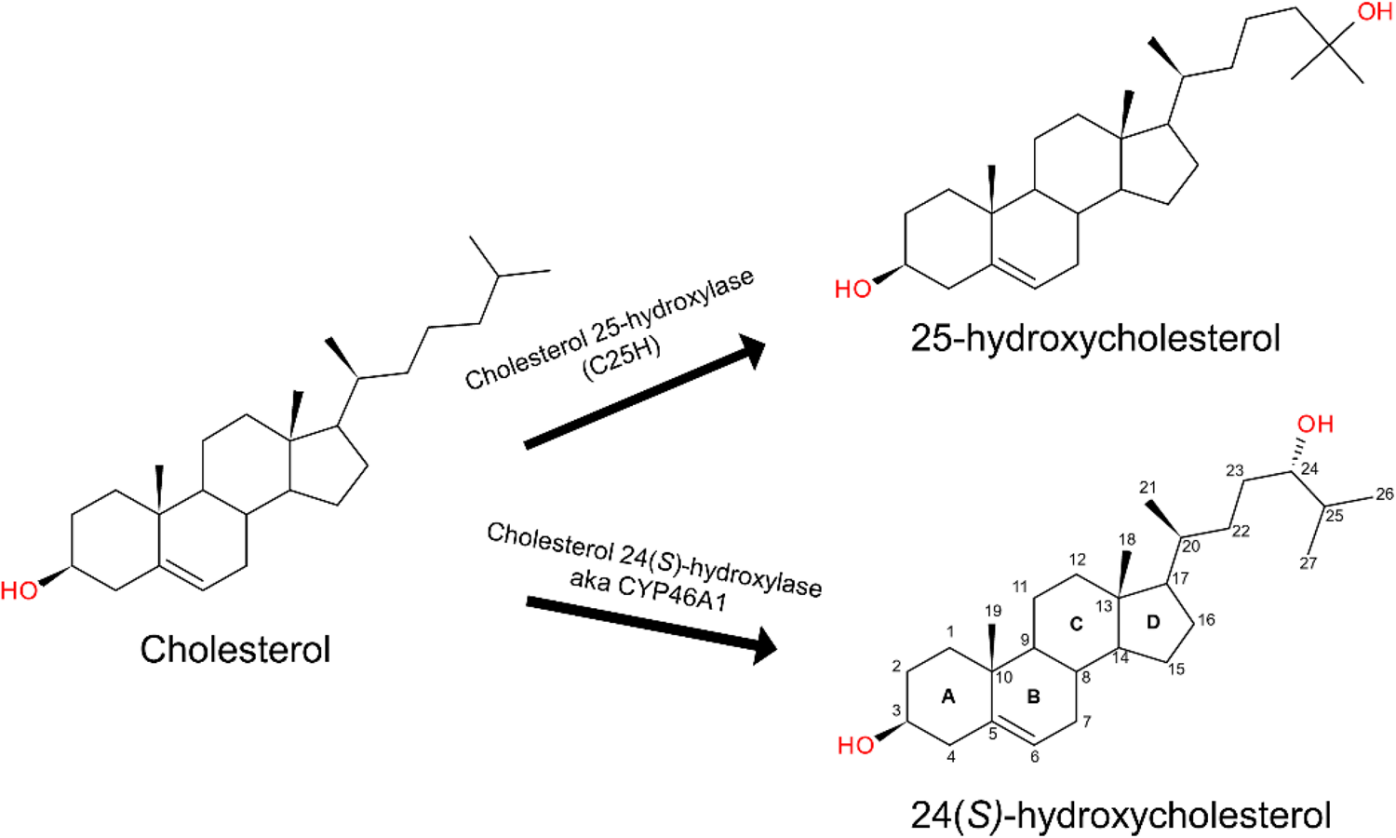
2D structures and metabolic pathways of cholesterol and its two metabolites. 25-hydroxycholetserol (25HC) and 24(S)-hydroxycholesterol (24HC) are produced by enzymatic metabolism of cholesterol cholesterol-25-hydroxylase (C25H) and cholesterol-24-hydroxylase also known as CYP46A1, respectively.

Oxysterols are oxygenated metabolites of cholesterol produced in the human body by enzymatic and non-enzymatic reactions^1,2^ (Fig. 1). They are biologically active and exert pleiotropic functions through multiple targets such as nuclear receptors, G protein-coupled receptors (GPCRs), regulatory or transport proteins (e.g., oxysterol binding protein), or by changing cell membrane properties^3–6^. In recent years, oxysterols such as 25-hydroxycholesterol (25HC) have been established to have important roles in immunity and inflammation, development, and many pathological conditions, including atherosclerosis, diabetes, Alzheimer’s disease, Parkinson’s disease, and cancer^7–15^. Their levels are significantly altered in many pathophysiological conditions, and some oxysterols are used as biomarkers of specific disease conditions. Recently, we uncovered a novel cellular mechanism of 25HC-mediated regulation of proinflammatory response^16^. We showed that 25HC triggers the production of proinflammatory mediators such as TNF and IL-6 by directly binding to αvβ3 and α5β1 integrins and activating the integrin-focal adhesion kinase (FAK) pathway. We also discovered that 25HC binds to a novel binding site (site II), distinct from the primary RGD-binding site (site I), where the extracellular matrix (ECM) ligands containing an Arg-Gly-ASP motif are known to bind. Site II is located at the interface between the β-propellor domain (of αv subunit) and the βI domain (of β3 subunit) and is placed at a distal side (approximately 6 Å) of site I. Site II was known to bind fractalkine (FKN), phospholipase A2 (sPLA-IIA), and CD40L^17^. The binding of 25HC at site II produced significant conformational changes in the specificity-determining loop (SDL) that seem to allosterically connect the two sites. The conformation of SDL has been shown critical for the α-subunit association specificity and heterodimer formation^18^. Most importantly, the specific conformation of SDL residues dictates the affinity and functional outcomes of ligands binding to the classical “RGD”-binding site^19–21^.

24-hydroxycholesterol (24HC), a structural isomer of 25HC, is also an oxygenated metabolite of cholesterol, catalyzed by the enzyme cholesterol 24-hydroxylase (also known as cytochrome P450 46A1, CYP46A1). 24HC is also called cerebrosterol since it plays an important role in maintaining cholesterol homeostasis in the brain^22^. It has also been implicated in several diseases, including Alzheimer’s, glaucoma, and obesity^22–26^. The conversion of the blood–brain barrier impermeable cholesterol to highly permeable 24HC and transport to the peripheral/systemic circulation is regarded as a major mechanism of cholesterol removal from the brain to maintain cholesterol homeostasis^26–30^. Remarkably, 24HC level in cerebral spinal fluid is considered a sensitive marker of the early stages of Alzheimer’s disease^31^. Advanced Alzheimer’s patients have a reduced level of 24HC in the plasma, reflecting marked destruction of the CNS and decreased expression of CYP46A1^32,33^.

It is still unknown whether integrins interact with 24HC and the role of such interaction during 24HC-mediated inflammatory response in non-neuronal cells. Especially since 24HC is detected in the circulation, it may trigger a proinflammatory response in non-neuronal immune cells like macrophages^28–30,34^. However, so far, no studies have investigated 24HC-dependent responses in macrophages.

In this study, we used a combination of in silico analysis, in vitro SPR binding assay, and *cell-based* assays with macrophages to delineate 24HC and integrin interactions for triggering a proinflammatory response in immune cells. Our in-silico studies comprising molecular docking, unbiased MD, and well-tempered metadynamics simulations revealed potential binding of 24HC to the integrin αvβ3 site II. Although 24HC is structurally similar to 25HC, surprisingly, as highlighted in the current study, we observed key differences in 24HC’s interaction with integrin αvβ3 compared to 25HC interactions reported previously. Subsequently, SPR binding assay showed direct interaction of 24HC with purified integrin αvβ3 with a relative binding affinity comparable to that of 25HC. Tumor necrosis factor-α (TNF) is a potent proinflammatory cytokine involved in regulating immunity/inflammation, programmed cell death, lipid metabolism, insulin resistance, and endothelial function^35–42^. Moreover, TNF triggers an inflammatory response that culminates in the development of a wide spectrum of hyper-inflammatory diseases like arthritis, sepsis, and pneumonia^35–42^. TNF is primarily produced from immune cells like macrophages, and therefore, we analyzed the role of TNF during the 24HC-mediated response in macrophages. Our in vitro study showed upregulation of 24HC converting enzyme CYP46A1 in macrophages treated with TNF. Furthermore, treatment of macrophages with 24HC also resulted in the robust production of TNF, illustrating the proinflammatory activity of 24HC in non-neuronal cells of immune origin (i.e., macrophages). In addition, blocking integrin-FAK-NFκB signaling by FAK- and NFκB-specific inhibitors significantly diminished 24HC-mediated proinflammatory responses in macrophages. Overall, this study identified integrin αvβ3 as a potential cell-surface receptor for 24HC, triggering a proinflammatory response in macrophages through the integrin-FAK-NFκB signaling pathway. Thus, this study highlights a yet unknown role of 24HC in triggering a proinflammatory response in immune cells like macrophages.

## Results

Our previously published study demonstrated that 25HC directly binds to “RGD”-binding αvβ3 and α5β1 integrins at site II and produces a proinflammatory response through the integrin-FAK-NFκB pathway. Being structural isomers, 24HC and 25HC possess identical physicochemical properties, and the only difference is in the position of the hydroxyl group at the alkyl chain (Fig. 1 and Supplementary Table S1). Thus, we hypothesized that 24HC would also bind to integrin αvβ3 and likely produce a proinflammatory response similar to 25HC. Therefore, we carried out a series of studies to investigate the potential interactions of 24HC with integrin αvβ3. First, we performed extensive molecular docking and molecular dynamics simulations to elucidate the potential binding mode and identify critical residue interactions. The results from these in-silico studies further guided us to perform in vitro studies in which we measured the binding affinity of 24HC to integrin αvβ3 using SPR and performed several functional assays with macrophages to determine the role of integrin-driven cellular signal transduction pathway involving FAK and NFκBin triggering the 24HC-mediated proinflammatory response.

### 24HC binds at site II of integrin αvβ3 in a distinct binding mode

**Figure 2:**
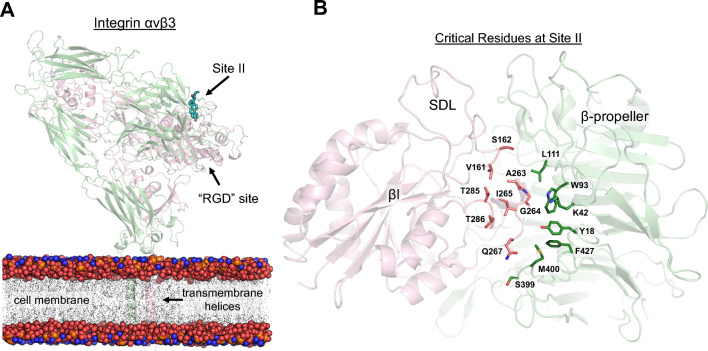
Oxysterols such as 25-hydroxycholesterol bind to integrin αvβ3 at site II, which is distinct from the primary RGD-binding site. (**A**) A full-length integrin αvβ3 (secondary structure representation) bound to an oxysterol (licorice representation) at site II is shown embedded in the lipid bilayer consisting of POPC and cholesterol. The αv and β3 subunits of integrin are shown in light green and pink colors, respectively. Site II, denoted by an arrow, is 6 Å away from the primary “RGD” binding site, where extracellular matrix proteins such as fibronectin are known to bind. The polar headgroups of the lipid bilayer are represented as the vdW surface model (nitrogen in blue, phosphate in orange, and oxygen in red colors), and the alkyl chains are shown as a transparent stick model. The extracellular domain is embedded in the bilayer through two transmembrane helices. (**B**) Site II is formed at the interface between the β-propeller domain (light green) of the αv subunit and the βI domain (light pink) of the β3 subunit. Critical binding site residues from each domain are shown in respective darker colors. The specificity-determining loop (SDL) connects site II with the primary “RGD” binding site.

**Figure 3:**
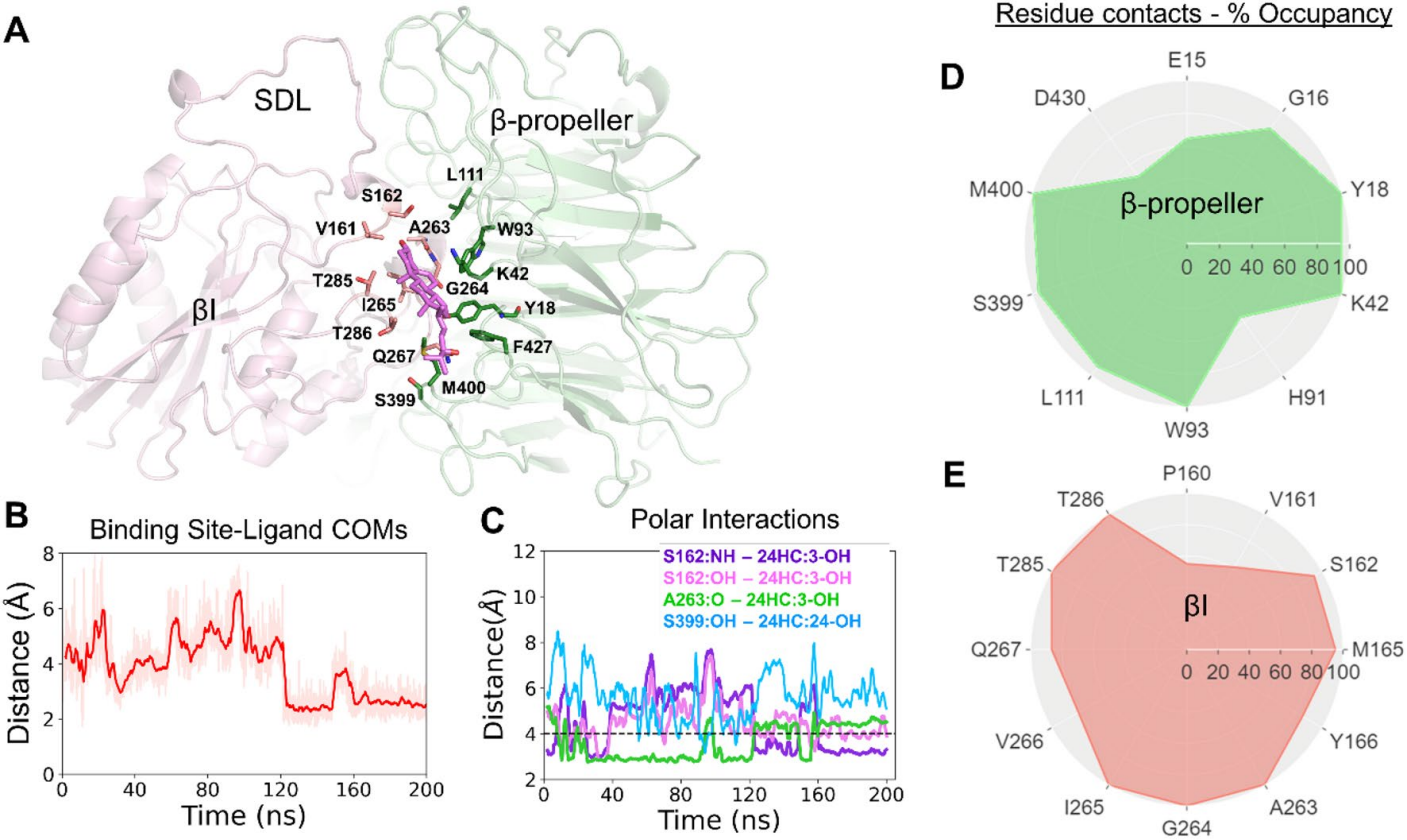
Molecular interactions of 24HC at site II of integrin αvβ3. (**A**) Molecular docking and MD simulations revealed that 24HC binds to integrin αvβ3 at site II in an orientation that is distinct from that of 25HC. In this orientation, the 3-OH group engages in polar interactions with S162 and A263 of the βI domain, and the 24-OH group is near S399 of the β-propeller domain. The two domains of integrin αvβ3 are shown as secondary structure representation, and the binding site residues are shown in licorice. (**B**) The distance between the center-of-mass (COM) of the binding site residues and COM of 24HC through the entire simulation time (200 ns) indicates the stability of the ligand within the binding site. (**C**) Major polar interactions between the two hydroxyl groups of 24HC and various binding site residues are tracked as distances between the interacting functional groups. (**D**–**E**) Radar charts showing the polar and hydrophobic interactions between 24HC and various binding site residues quantified as % occupancy, the fraction of the simulation time during which 24HC is within 5 Å of the listed residues from the β-propeller domain (light green), and the βI domain (light pink), respectively.

To determine the potential binding mode(s) of 24HC at site II of integrin αvβ3, we performed a series of molecular docking simulations using multiple ligand-placement methods and scoring functions (see “Materials and methods” section for details). The full-length integrin αvβ3 structure (PDB ID 6AVQ) containing the extracellular domains and two transmembrane helices was used for the docking and MD simulations. The modeling of transmembrane helices and construction of the full-length integrin αvβ3 structure, including fixing the missing residues/atoms, were described in the “Materials and methods” section. Site II is formed at the interface between the β-propeller domain of the αv subunit and the βI domain of the β3 subunit (Fig. 2). The binding site is characterized by the presence of hydrophobic and polar residues from the β-propeller domain (L111, W93, K42, Y18, F427, M400, S399) and the βI domain (S162, A263, V161, I265, T285, T286, Q267). Molecular docking was performed using an induced-fit docking protocol in MOE, in which the side chains of the binding site residues were allowed to be flexible and optimized after ligand placement at the final refinement stage. Each pose was scored based on a scoring function, and top-scoring poses with meaningful interactions were manually chosen for subsequent all-atom MD simulations^43^. Figure 3A shows one of the top-scoring docked poses of 24HC at site II of integrin αvβ3. The 3-OH polar group of 24HC makes H-bond interactions with the sidechain –OH group of S162 and backbone –NH group of A263 from the βI domain. The 24-OH group makes favorable electrostatic interactions with the sidechain and backbone polar functional groups of residues S399 and M400 from the β-propeller domain. The steroidal ring of 24HC makes extensive hydrophobic contacts with the interface residues V161, I265, T285, and T286 (from the βI domain) and L111, W93, K42, Y18, and F427 (from the β-propeller domain). Surprisingly, this binding pose of 24HC is distinct from the putative binding pose of 25HC, reported previously^16^. The reported binding pose of 25HC was such that the 3-OH group engaged with S399, whereas the 25-OH group interacted with S162 and A263. Although the docking simulations produced similar orientations for 24HC, the subsequent 200 ns long MD simulations confirmed that only the reverse orientation is stable within the binding site.

Next, the proper orientation of full-length integrin αvβ3-24HC complexes (top-scoring poses) in the plasma membrane was obtained using OPM (Orientation of Protein in Membrane) webserver^44^, and the protein–ligand complexes were embedded in a model membrane made up of 1-palmitoyl-2-oleyl-glycero-3-phosphocholine (POPC) lipid and 30% cholesterol. The integrin αvβ3-24HC complexes embedded in the membrane were solvated with explicit water and subjected to 200 ns length all-atom MD simulations. Each binding pose was simulated in three replicas (See the “Materials and methods” section for details). The stability of 24HC within the binding site and critical residue interactions for each pose was analyzed using the trajectories obtained from the MD simulations.

### Stability of the integrin αvβ3-24HC complex

Although the molecular docking simulations predicted two distinct binding poses (one is inverted of another), the subsequent MD simulations revealed that 24HC was stable within the binding site in only one orientation. In the opposite orientation, which is similar to the binding pose of 25HC, 24HC came off from the binding pocket in all three simulations. In contrast, in the inverted binding orientation, 24HC engaged in multiple stable polar, H-bond, and hydrophobic interactions with the binding site residues. The distance measured between the center-of-mass (COM) of the heavy atoms of 24HC and the COM of the binding site residues through the entire simulation time (200 ns) remained around and below 4–5 Å, indicating the lasting interactions and stability of the ligand within the pocket (Fig. 3B). During the first 100 ns of the simulations, the COM distance fluctuated between 4 and 5 Å as 24HC slightly shifted from its docked position within the binding site, adjusting the interactions between the 3-OH group and the backbone and sidechain functional groups of residues S162 and A263.

The stability of the integrin-24HC complex was assessed using the root mean squared deviation (RMSD) values of the interacting species (Fig. S2A). The RMSD value of the receptor was calculated using the backbone and sidechain heavy atoms after removing all rotational and translational motion of the protein and the initial docked complex as a reference frame. Time evolution of the integrin RMSD value through the entire simulation time showed that the RMSD plateaued around 4–6 Å after 50 ns. A significant conformational change in SDL was observed around ~ 30 ns with the RMSD difference of ~ 10 Å. A slight rearrangement of 24HC occurred around 20–30 ns, resulting in ~ 5 Å RMSD change, after which the ligand stabilized within the binding site for the remaining simulation time.

When bound, 24HC is sandwiched at the hydrophobic groove formed between the βI (β3 subunit) and β-propeller (αv subunit) domains of integrin αvβ3. The stability of the integrin-24HC complex and favorable interactions of 24HC with the site II residues were further assessed by the extent of solvent-accessible surface area (SASA) of the ligand buried within the site (buried surface area, BSA). The total SASA for 24HC is approximately 680 Å^2^ (Fig. S2B). Throughout the simulation time, more than 45–50% of 24HC’s SASA is buried in the site, with the BSA value of 24HC increasing from ~ 300 Å^2^ to nearly ~ 400 Å^2^ as the simulation progressed. In this preferred binding orientation, two methyl groups on the steroidal ring rough surface (β-surface) of 24HC were oriented toward the β-propeller domain.

### Critical interactions of 24HC with the binding site residues of integrin αvβ3

The molecular interactions between 24HC and the site II residues of integrin αvβ3 comprised extensive hydrophobic and electrostatic interactions, including several H-bonds. As shown in the figure (Fig. 3A and C), the 3-OH group of 24HC made a strong H-bond interaction with the backbone N-atom of S162 as the hydrogen bond distance and angle were below 3.5 Å and ~ 125°, respectively. A slight rearrangement of 24HC from its docked pose occurred around ~ 30 ns, which led to the formation of strong H-bond interactions between the 3-OH and the sidechain hydroxyl group of S162 and the backbone carbonyl oxygen of A263. These interactions remained intact and lasted until 200 ns. Although the H-bond interaction of the 3-OH group switched back and forth between A263 and S162, the 3-OH group remained engaged in strong H-bond interactions with site II. The bond distance between the backbone carbonyl oxygen of A263 and the 3-OH group remained below 3.5 Å (Fig. 3C), and the bond angle remained around 150°, indicating strong H-bond interactions. On the other hand, the 24-OH group made lasting electrostatic interactions with the sidechains of residues S399 and M400 from the β-propeller domain. The extensive polar and hydrophobic interactions of 24HC (the two hydroxyl groups, the steroidal ring, and the alkyl chain) with various residues from the β-propeller and βI domains at site II were also quantified as contact frequency (% occupancy) as illustrated in the radar plots (Fig. 3D and E, respectively). The contact frequency indicates the fraction of the simulation time (%) during which a particular residue is within the cutoff distance of 5 Å, from 24HC.

The radar plots show residues that have a contact frequency of at least 50% and above from the β-propeller (E15, G16, Y18, K42, H91, W93, L111, S399, M400, and D430) and βI (P160, V161, S162, M165, Y166, A263, G264, I265, V266, Q267, T285, and T286) domains. From the β-propeller domain, Y18, K42, and W93 form a triad engaging in alkyl-alkyl, aryl–alkyl, and cation-π interactions with 24HC. The β-propeller residues Y18, K42, W93, S399, and M400, had more than 95% contact occupancy engaging in interactions with the steroidal ring atoms as well as the alkyl chain atoms. Specifically, Y18, K42, and W93 had close contact with C5-C19 and C21 atoms of the rings B, C, and D of 24HC (See Fig. 1 for the atom numbers), whereas S399 and M400 were mostly in contact with the alkyl chain C20-C27 atoms. Residues such as G16 and L111 had ~ 80% occupancy, and E15, H91, and D430 had 40–60% occupancy, respectively. The βI domain residues S162, M165, A263, G264, I265, T285, and T286 had more than 95% occupancy. The residues P160, V161, Y166, V266, and Q267 had contact occupancies ranging from 40 to 80%. A263 and S162 were mainly engaging in H-bond interactions with 3-OH. Also, the backbone N-atom of G264 appeared to make electrostatic interactions with 3-OH with a distance of ~ 5 Å. Stable and lasting hydrophobic interactions were seen between the sidechain methyl groups of M165, I265, T285, and T286 and C1-C12 atoms of the steroidal rings A and B of 24HC. In addition, the sidechain atoms of T286 made contact with the rings C and D (C13-C17) as well as the alkyl chain (C21) atoms.

### The binding orientation of 24HC is further validated by well-tempered metadynamics

**Figure 4:**
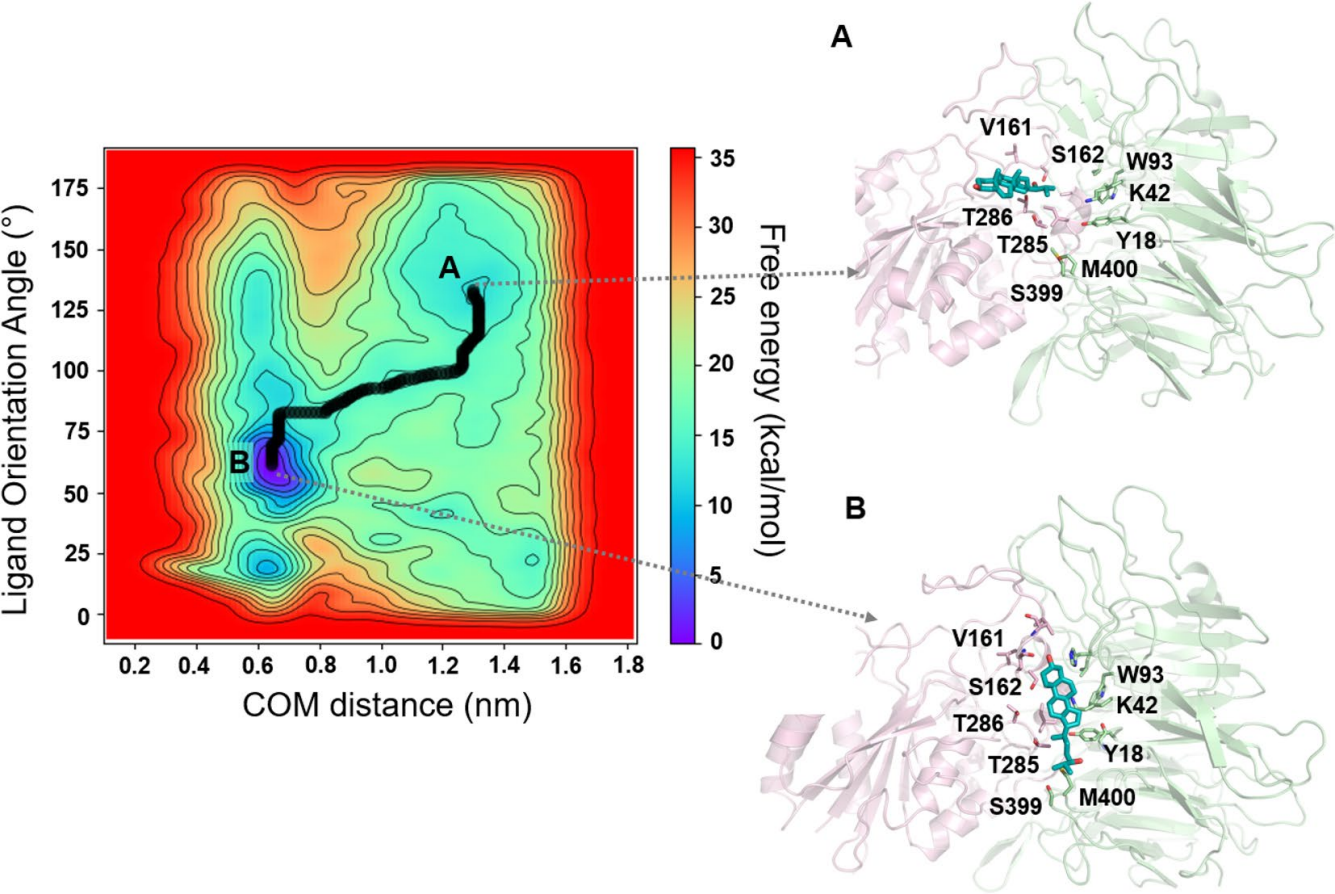
The free energy surface (FES) for 24HC’s access and binding to the integrin αVβ3 site II. The FES was characterized by two collective variables: (1) the distance between the center-of-mass (COM) of the ligand and COM of the binding site residues in nm (X-axis), and (2) the orientation angle of the ligand, defined as the angle between 3-OH and C23 of 24HC and the backbone carbon atom of Ile265 in degrees (Y-axis). The minimum energy path of 24HC’s access to the binding site is given as black bold connected points. **A** and **B** represent one of the intermediate states and a final bound state, respectively.

Surprisingly, the potential binding orientation of 24HC at site II, as predicted by the docking and MD simulations, is distinct and in reversed orientation from that of 25HC. To further validate this binding orientation, we ran multiple association simulations using well-tempered metadynamics (WT-metaD), an enhanced sampling technique used to predict the access path and binding orientation of ligands to its target site within a reasonable timescale (mostly < 50 ns). 24HC was placed randomly at approximately 30–40 Å away from the binding site residues in the aqueous phase and was allowed to reach the site by taking energetically favorable paths without constraining their orientation, conformation, or access trajectory. The free energy surface (FES) of the ligand access and binding path was characterized by two collective variables: (1) the distance between the center-of-mass (COM) of 24HC and COM of the binding site residues in nm (X-axis), and (2) the orientation angle of the ligand, defined as the angle between 3-OH and C23 of 24HC and the backbone carbon atom of Ile265 in degrees (Y-axis), as shown in Fig. 4. In 14 out of 24 simulations (~ 60%), 24HC reached the binding site and assumed the reversed binding orientation as predicted by the docking and MD simulations. In the rest of the simulations, 24HC assumed a binding orientation similar to that of 25HC. It should be noted that only the reverse orientation was found to be stable during MD simulations for the entire 200 ns. During the association simulations, initially, 24HC approached the binding site with its 24-OH group pointing toward the site, and the molecule was mostly oriented perpendicular to the plane of the site (Fig. 4A). As 24HC moved closer to site II, the molecule gradually flipped upside down with its 3-OH group positioned near the binding site while the 24-OH group was still exposed to the surrounding water molecules. Finally, 24HC entered the binding site facilitated by multiple polar and nonpolar interactions with site residues (Fig. 4B) and assumed its final pose that is similar to one predicted by molecular docking and MD simulations. The free energy surface indicates that the final bound conformation of 24HC is energetically more favorable than the intermediate states. We performed similar association simulations for 25HC in three replicates. In all three simulations, 25HC was bound to the site in reverse orientation, as reported by us in our previous study. The FES of a representative simulation is shown in Supplementary Fig. S6.

### 24HC-induced conformational change in the SDL loop

**Figure 5:**
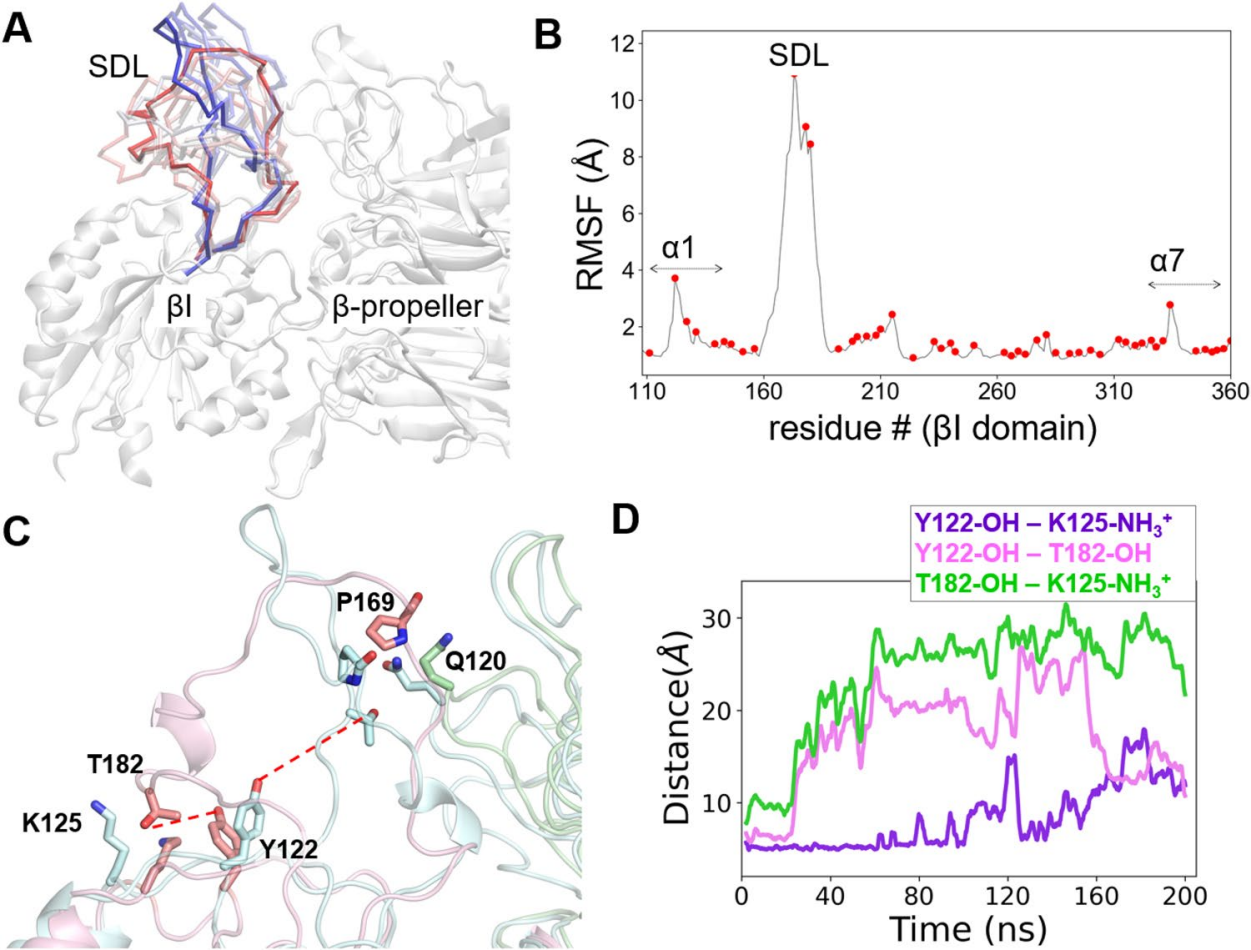
24HC produces significant conformational changes in the specificity-determining loop (SDL) in the βI-domain of integrin αvβ3. (**A**) SDL undergoes extensive conformational changes during 200 ns MD simulations. The time evolution of the entire loop (residues 158–190) is shown at various time intervals from the start (red color) to the end (blue color). (**B**) The root-mean-square-fluctuations (RMSF) indicate the extent of conformational changes in the βI-domain observed during the simulations. In addition to SDL, both α1 and α7 helices undergo moderate fluctuations. (**C**–**D**) 24HC binding disrupts H-bond networks within the SDL residues and between SDL and the β-propeller domain. SDL is shown at the start and end of the simulation in pink and cyan colors, respectively. The H-bonds between Y122 and T182 and T182 and K125 broke after 30 ns. The H-bond between Y122 and K125 broke after around 100 ns. The H-bond between the residues P169 from SDL (βI) and Q120 from the β-propeller domain quickly moved apart from 2.9 to 16.6 Å. The β-propeller domain (with Q120) is shown at the start and end of the simulation in green and light blue colors, respectively.

Integrin αvβ3 is a heterodimer made up of αv and β3 subunits. In the βI-domain of the β3 subunit, a loop region made of a set of residues (from 158 to 190) called specificity determining loop (SDL). The SDL loop has been shown to be critical for the binding of “RGD” ligands and dictate specific α-subunit association through the conformational changes^45^. The binding of 24HC at site II produced a significant conformational change in the SDL loop, resulting in the RMSD change of ~ 10 Å (Fig. S2A) and an increase in the solvent-accessible surface area (SASA) by more than 200 Å^2^. As shown in Fig. 5B, SDL had a greater mean residue fluctuation (RMSF ~ 10 Å) than the rest of the βI domain. Considering SDL’s significant conformational change, we monitored whether there was a variation in the secondary structure content of the SDL loop due to 24HC binding. The C-alpha atoms of the SDL loop residues were selected to calculate the secondary structure content using the VMD STRIDE program^46^. Surprisingly, no changes were observed in the secondary structure content along the entire trajectory (Fig. S3B). As shown in Fig. S3C, 20% of the SDL loop remained 3_10_-helix, 70% in random coil, and 10% turn. After ~ 40 ns into the simulation, the SDL loop moved away from the β-propeller and towards the βI domain, resulting in a disruption of the H-bond network between the RGD-binding site residues Y122 and K125 and the SDL residue T182. Initially, the sidechain –OH group of T182 was engaged in polar H-bond interactions with the sidechain phenyl –OH group of Y122 and the sidechain charged amino group of K125. These interactions were broken within 40 ns into the simulation resulting in the movement of the loop (Fig. 5C–D). The electrostatic interactions between the sidechain amino group of K125 and the sidechain –OH group of Y122 (distance < 5 Å increases to > 10–15 Å) become weaker after 80 ns. However, the strong H-bonds between the backbone amino and carbonyl groups of Y122 and the backbone carbonyl group of S213, and the backbone amino group of K125, respectively, remained intact and below 4 Å throughout the simulation time (Fig. S4A). Both Y122 and K125 are key residues in the integrin βI domain that recognizes RGD ligands such as fibronectin^47^.

The polar interactions between the SDL loop residue P169 and N120 from the β-propeller domain undergo significant changes due to the conformational change in SDL (Fig. S4A). Further, the interaction distances between the site II residue S162 and A263, and H113 are well above 4 Å, similar to the distances observed in the active receptor bound to fibronectin (Fig. S4A). In addition, the interactions of the sidechain Y122 with R214 as well as M180 seem to vary during the simulation time. However, the interactions observed between the backbone carbonyl group of M180 and the side chain –OH and backbone amino groups of T182 remain relatively stable throughout the simulation time.

### Outside-in activation conformational dignatures

Integrins are cell surface receptors that transmit signals bidirectionally across the cell membrane^48^. The RGD-motif-containing ligands such as fibronectin and vitronectin bind to the integrin ectodomain at site-I (Fig. 1A), produce the outside-in activation and transmit signals across the cell membrane through a repertoire of conformational changes. Ligand binding to the integrin leads to distinct conformational changes in the βI domain, three metal-binding sites and associated loops, and α1- and α7-helices. Here, we highlight some notable structural changes in the integrin αVβ3 due to 24HC binding at site II. The RMSD of α1 and α7 helices increased by ~ 3–4 Å after approximately 100 ns (Fig. S5A). However, the solvent-accessible surface area (SASA) of those residues remained unchanged in the range of 1700–1800 Å^2^, indicating the absence of any major structural changes (Fig. S5B). The angle between the βI and hybrid domains ranged between 60 and 65°, and there is an increasing trend observed in the angle during the simulation time (Fig. S5C). Additionally, the center-of-mass (COM) distance between the Thigh and Psi domains remained around 43 Å (Fig. S5D). In addition, we monitored the dynamics of the αV and β3 transmembrane helices. The COM distance between the TM helices did not change much and remained below 12 Å. It should be noted that the timescale of the MD simulation in this study, i.e., 200 ns, may not be sufficiently long to observe all the outside-in activation mechanisms in a definitive manner.

### Surface plasmon resonance (SPR) analysis to determine the binding affinity of 24HC to integrin αvβ3

**Figure 6:**
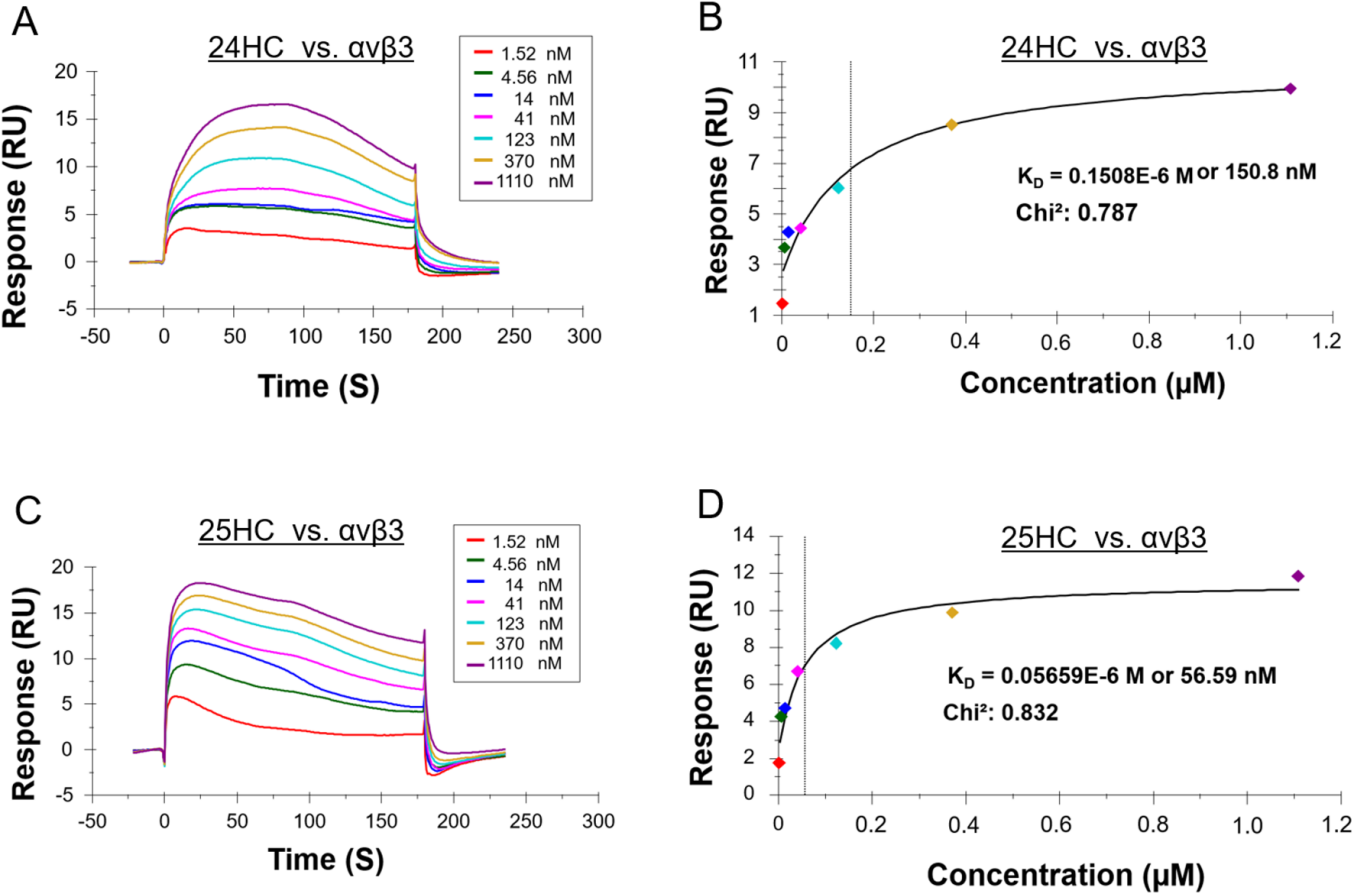
The binding affinities of 24HC and 25HC to integrin αvβ3 were determined by surface plasmon resonance (SPR). (**A**–**B**) 24HC and (**C**–**D**) 25HC. Increasing concentrations of the ligand (as indicated in the figures) were injected into both the integrin-immobilized and control blank surface of the CM5 chip, as described in “Materials and methods” Section. The analyte injection was terminated at 180 s and allowed to dissociate in the running buffer for 600 s. Representative SPR data were quantified using the steady-state equilibrium binding model to calculate the affinity constant (*K*_D_), as shown in the curve-fitting graphs.

Our molecular docking and MD simulations revealed the potential binding of 24HC to integrin αvβ3 at site II in a distinct binding mode as compared to 25HC. To validate the molecular interactions and determine the relative binding affinities of 24HC and 25HC to integrin αvβ3, we performed surface plasmon resonance (SPR) analyses using a Biacore S200 in which purified integrin αvβ3 protein was immobilized on a carboxy-methylated dextran coated chip. The SPR sensorgrams obtained by injecting the increasing concentrations of the ligands were used to determine the affinity constants (K_D_) using the steady-state equilibrium binding model. As predicted by the in-silico studies, the interaction of 24HC with αvβ3 integrin was specific since 24HC bound to αvβ3 integrin at a sub-micromolar affinity with the K_D_ value of 150.8 nM (Fig. 6A–B). As previously reported by us, 25HC also binds to αvβ3 integrin with a higher affinity with a K_D_ value of 56.59 nM (Fig. 6C–D). Interestingly, the binding affinity of 25HC was three times greater than that of 24HC, suggesting that the molecular interactions between 25HC and integrin αvβ3 are notably stronger compared to the interactions of 24HC with integrin αvβ3. This difference in affinity could be attributed to the distinct and opposite binding orientations by which these two molecules bind to site II of αvβ3 integrin. Nevertheless, our results showing the sub-micromolar affinity (K_D_) values obtained for both 24HC and 25HC indicate notable and specific binding of these molecules for integrin αvβ3.

### Cholesterol 24-hydroxylase (CYP46A1) is induced by TNF in macrophages, the production of which is triggered by 24HC

**Figure 7:**
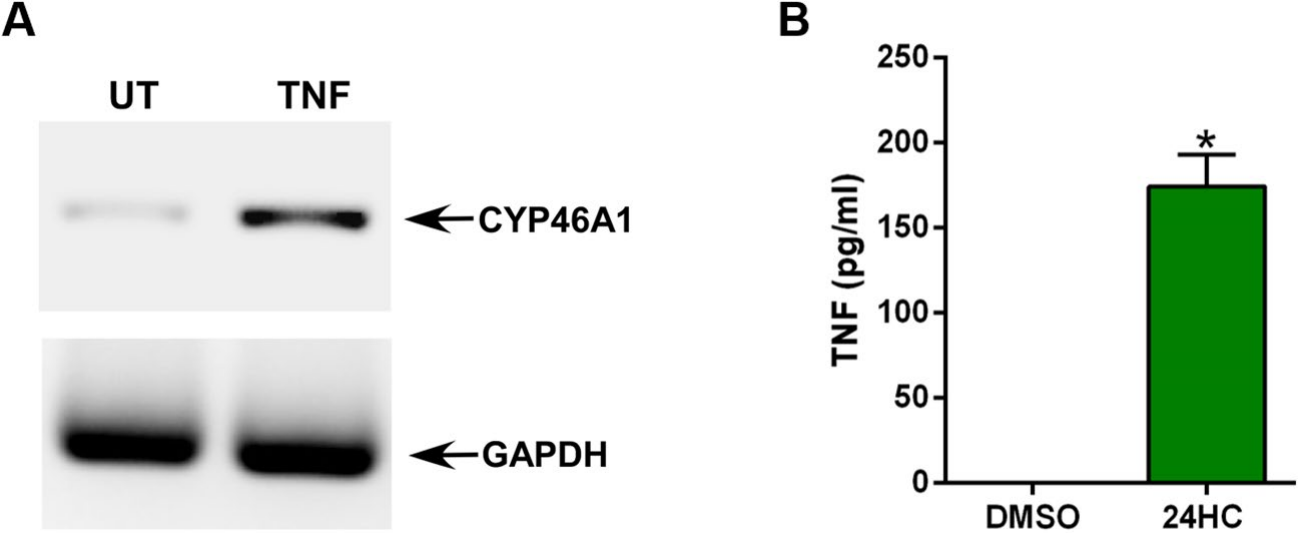
24HC converting enzyme CYP46A1 is induced by Tumor Necrosis Factor-alpha (TNF) in macrophages, and 24HC activates a proinflammatory response in macrophages. (**A**) RT-PCR analyses of CYP46A1 expression in RAW 264.7 macrophages treated with TNF (100 ng/ml) for 2 h. (**B**) RAW 264.7 macrophages were treated with either DMSO (vehicle) or 24HC (50 µM) for 16 h. TNF production was analyzed by ELISA. The ELISA values are represented as mean ± standard deviation (n = 16 technical replicates from two independent experiments). *p ≤ 0.05 using Student’s t-test.

Molecular docking and SPR data suggested that integrin signaling may play a role in the 24HC-mediated response. However, so far, no studies have been reported demonstrating 24HC-mediated response in non-neuronal cells, and the functional role of 24HC in immune cells like macrophages is yet unknown. These studies are important since 24HC in the circulation may trigger a proinflammatory response in non-neuronal immune cells like macrophages^28–30,34^. TNF, a potent proinflammatory cytokine promoting various diseases, including hyper-inflammatory diseases (e.g., arthritis, sepsis, pneumonia)^35–42^ is primarily produced from immune cells like macrophages. We previously showed that the 25HC-generating enzyme C25H is induced in macrophages by proinflammatory cytokines like TNF^16^. Additionally, 25HC triggered a proinflammatory response in macrophages by producing TNF, and this response was mediated via the integrin-FAK-NFκB signaling pathway^16^. Therefore, we first examined whether the 24HC converting enzyme CYP46A1 is induced in macrophages by a proinflammatory agent like TNF. To study CYP46A1 expression, we treated RAW 264.7 macrophages with TNF, followed by PCR analysis to detect CYP46A1 expression. TNF treatment led to robust induction of CYP46A1 expression in macrophages (Fig. 7A), demonstrating that macrophages can induce CYP46A1 expression. Next, we investigated whether 24HC triggers a proinflammatory response in macrophages. Treatment of RAW 264.7 macrophages with 24HC resulted in the production of proinflammatory cytokine TNF (Fig. 7B). Thus, proinflammatory induction of CYP46A1 by TNF and production of TNF by 24HC suggests that 24HC may be involved in amplifying TNF-mediated proinflammatory response, a mechanism that we recently demonstrated with another oxysterol 25HC^49^. Importantly, our results highlighted the role of 24HC in non-neuronal cells, particularly its involvement in the proinflammatory immune response in macrophages.

### FAK-NFκB signaling is essential for 24HC-mediated proinflammatory response in macrophages

**Figure 8:**
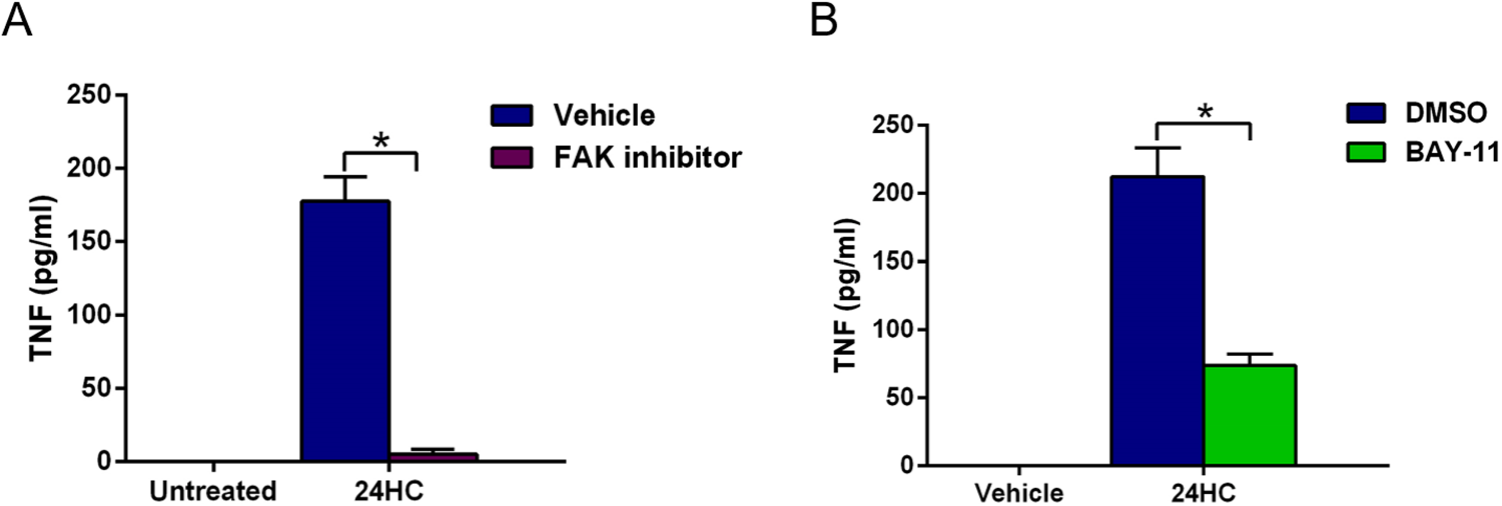
Integrin-FAK-NFκB signaling is essential for 24HC-mediated proinflammatory response in macrophages. (**A**) FAK is required for 24HC-mediated proinflammatory response. RAW 264.7 macrophages were treated with 24HC (50 µM) for 16 h in the presence of either DMSO (vehicle control) or FAK inhibitor PF-431396 (5 mM). TNF secretion was analyzed by ELISA. (**B**) NFκB is required for 24HC-mediated proinflammatory response. RAW 264.7 macrophages were treated with 24HC (100 µM) for 16 h in the presence of either DMSO (vehicle control) or NFκB inhibitor BAY-11 (1 μM). TNF secretion was analyzed by ELISA. The ELISA values are represented as mean ± standard deviation (n = 14–24 technical replicates from two–three independent experiments). *p ≤ 0.05 using Student’s t-test.

Focal Adhesion Kinase (FAK) is a key adaptor protein in the integrin signaling pathway^50,51^. Integrin signaling cannot operate in the absence of functional FAK protein^50,51^. Since our molecular docking and SPR data suggested the role of the integrin pathway in 24HC-mediated response, we investigated whether FAK is required for 24HC-dependent proinflammatory response in macrophages. We assessed the production of proinflammatory cytokine TNF from 24HC treated RAW 264.7 macrophages in the absence (control) or presence of a FAK inhibitor, PF-431396. A robust proinflammatory response was detected in control cells treated with 24HC (Fig. 8A). However, such a response was abrogated following treatment of cells with the FAK inhibitor. Thus, 24HC mediated response in macrophages is FAK-dependent, which supports the plausible 24HC-integrin interactions as revealed by the molecular docking and SPR analyses.

NFκB is a key proinflammatory transcription factor involved in relaying integrin-FAK signaling^52^. Activation of the integrin-FAK pathway results in NFκB activation and its subsequent targeting of the nucleus, where it transactivates the expression of proinflammatory genes, including TNF. Since the FAK pathway is crucial for 24HC mediated response (Fig. 8B), we next evaluated the role of NFκB during this process by blocking NFκB activation in 24HC treated RAW 264.7 macrophages with widely used NFκB inhibitor BAY-11. Blocking NFκB activation led to a significant reduction in TNF production following 24HC treatment of macrophages. This result demonstrated that 24HC activates the integrin-FAK-NFκB pathway for proinflammatory response in macrophages.

## Discussion

Hydroxylated forms of cholesterol, such as 25HC and 7α, 25-dihydroxycholesterol have been shown to play essential roles^4,10,53^ in multiple pathophysiological processes, including innate and adaptive immune responses. Recently, using combined in vitro and in silico studies^16^, we demonstrated that 25HC directly binds to integrins with high affinity, activates the FAK signaling pathway, and enhances proinflammatory cytokines production through NFκB activation.

24HC is implicated in multiple neurodegenerative diseases, including Alzheimer’s and glaucoma, a progressive and irreversible blinding neuropathy^22,26,52–55^. Decreased levels of 24HC have been found in obese and metabolic syndrome patients^23^. 24HC has been shown to produce a proinflammatory response in human neuroblastoma cells (or AD patients) via Toll-like receptor-4/cyclooxygenase-2/membrane bound prostaglandin E synthase (TLR4/COX-2/mPGES-1)^15,56–58^. Also, 24HC significantly induced the expression of proinflammatory cytokine interleukin 8 (IL-8) in neuroblastoma cells. Interestingly, another study reported a 2-fold enhancement of integrin β1 expression by 24HC, suggesting a possible role of integrins during 24HC-dependent response^59^.

The majority of 24HC-related studies were performed in neuronal cells. The role of 24HC in non-neuronal cells is still lacking. Especially, immune cells like macrophages have not been used as a potential target for a 24HC-mediated response. In this study, we demonstrate that, like 25HC, 24HC also activated the FAK signaling pathways resulting in the enhanced production of proinflammatory TNF from macrophages. Interestingly, our docking and MD simulation studies revealed that 24HC binds to site II of integrin αVβ3 in a binding orientation distinct from 25HC. In our previously published study, 25HC has been shown to assume an upright orientation in which its 3-OH and 25-OH groups are near S399 and A263, respectively (Fig. 3A). Specifically, the 25-OH group engaged in strong and lasting H-bond interactions with the backbone carbonyl oxygen of A263 and the sidechain –OH or backbone –NH groups of S162 from the specificity-determining loop (SDL). Notably, the switching of interactions between 25-OH and the site II residues A263 and S162 seemed to trigger the conformational changes in SDL. The 3-OH group of 25HC engaged in moderate to strong electrostatic interactions with the sidechain –OH group of S399. In contrast, 24HC assumes a reverse orientation with its 3-OH and 24HC groups positioned near S162/A263 and S399, respectively. Most importantly, the H-bond to A263 through its 3-OH was more stable than the one observed with 25-OH from 25HC. Similar to 25HC, the polar interactions of the 24-OH group of 24HC with S399 were more electrostatic interactions throughout the simulation, with an average distance of around ~ 5 Å (Fig. 3C). In addition to polar interactions by two –OH groups, the steroidal moiety of 25HC has been shown to engage in nonpolar interactions with residues from both βI (Y18, K42, W93, L111, and M400) and β-propeller (V161, M165, A263, I265, Q267, V266, T285, and T286) domains. Similarly, 24HC has been shown to engage in stable and lasting hydrophobic interactions with these residues from both domains (Fig. 3D and E).

The surprisingly distinct orientation of 24HC in comparison to 25HC was further validated by well-tempered metadynamics (WT-metaD) simulations in which the ligands were allowed to reach the binding sites using two geometry-based collective variables. The distance between the COMs of the ligand and the binding site residues, and the orientational angle of the ligand, together, characterized the free energy of access and binding of 24HC to the receptor. Notably, in most simulations, 24HC was consistently bound to the receptor in the reverse orientation, as predicted by the docking and MD simulations. WT-metaD technique has been successfully applied to predict the access paths and binding modes of many GPCR ligands. For example, in our recently published studies, we have successfully used the WT-metaD technique in recreating the experimentally determined binding modes of antiasthma agents such as salmeterol, formoterol, and salbutamol to the β2-adrenergic receptor and for a negative allosteric modulator, ORG27569, of the cannabinoid CB1 receptor^60,61^. Therefore, we believe that 24HC and 25HC likely recognize site II of integrin αVβ3 in the binding modes, as predicted in this study. Although the binding orientations and residue interactions are distinct for 24HC and 25HC, the conformational changes produced by these ligands in the specificity-determining loop followed a similar pattern (Fig. 5A–B). Both ligands appear to disrupt the H-bond network between residues K125, Y122, and T182 (Fig. 5C and D). In addition, the electrostatic interactions between P169 of βI and Q120 of β-propeller domains were disrupted in similar ways, resulting in an increase of > 200 Å2 in the solvent-accessible surface area of the loop residues. As the SDL loop bridges the integrin site-I and site II, any significant conformational changes in the SDL loop induced by ligands bound at site II would likely affect the binding of RGD ligands at site I. This observation is intriguing and thus opens up the possibility that site II could be used to design allosteric modulators.

The potential binding of 24HC to integrin αVβ3 was further confirmed by surface plasmon resonance (SPR) studies using increasing concentrations of the ligand using a Biacore S200 sensor chip. As per the steady-state equilibrium model, the dissociation constant (K_D_) for 24HC was determined to be 150.8 nM. Although we reported a K_D_ value of 10.9 nM for 25HC in our earlier study carried out using Biacore 3000, we repeated the SPR experiments determining the binding affinity for 25HC again to account for any errors introduced by instruments, reagents, or personnel. Accordingly, the new K_D_ value of 56.59 nM was determined for 25HC, in consistent with our previous value. The three-fold difference in the binding affinity values between 24 and 25HC may stem from the fact that these ligands bind to site II in two distinct binding orientations, resulting in varied residue interactions.

24HC has been detected in the circulation/plasma^28–30,34^. For example, age-related macular degeneration (AMD) results in elevated levels of circulatory 24HC^28–30,34^. Moreover, several neurological and metabolic conditions (e.g., Alzheimer’s, glaucoma, obesity) affect plasma 24HC concentration^23–26,28–30,34^. Thus, 24HC levels in circulation during various pathophysiological conditions may regulate cellular immune homeostasis. Specifically, modulation of inflammatory immune response may occur during varying 24HC levels in the circulation. Macrophages are major immune cells involved in promoting a proinflammatory response that culminates in tissue inflammation. Thus, we examined whether 24HC can regulate proinflammatory response in macrophages. TNF, a potent proinflammatory cytokine promoting various diseases, including hyper-inflammatory diseases (e.g., arthritis, sepsis, pneumonia), is primarily produced from immune cells like macrophages^35–42^. Our in vitro studies with macrophages revealed that TNF treatment of macrophages resulted in an increased expression of CYP46A1, a microsomal enzyme involved in the conversion of cholesterol to 24-(S)-hydroxycholesterol (Fig. 7). Furthermore, 24HC-induced proinflammatory response since we detected the release of TNF from 24HC-treated macrophages. FAK is the critical integrin adapter molecule involved in relaying integrin-mediated cellular signaling^50,51^. In that regard, we show that 24HC-mediated proinflammatory response is mediated by FAK- NFκB signaling since TNF production was abrogated in cells treated with FAK and NFκB inhibitors (Fig. 8). Thus, we have identified 24HC as a proinflammatory molecule that is not only induced by proinflammatory cytokine TNF, but 24HC can also trigger a proinflammatory response by virtue of producing TNF from macrophages. This interplay between TNF and 24HC may play a key role in amplifying inflammatory response via autocrine/paracrine mechanisms.

## Conclusions

Oxysterols have been shown to participate in an increasing number of pathophysiological processes, including their involvement in innate and adaptive immune responses. In this study, we show that 24HC, a structural isomer of 25HC, produces a proinflammatory response in non-neuronal cells such as macrophages. The results from in silico, SPR, and in vitro cell-based studies suggest that, like 25HC, 24HC directly binds to integrin αVβ3 at site II, which is distinct from the primary RGD-binding site. However, the binding orientation and residue interactions of 24HC appear to be different from that of 25HC, likely contributing to its comparatively lower binding affinity. Notably, 24HC produced significant conformational changes in the specificity-determining loop (SDL), which connects site II to the RGD-binding site. These conformational changes may likely alter the molecular recognition of integrin αVβ3 of its extracellular matrix ligands, such as fibronectin, and affect their functional response. Furthermore, our in vitro studies with macrophages show that the proinflammatory cytokine, TNFα, induced the expression of cholesterol 24-hydroxylase (CYP46A1), the enzyme involved in converting cholesterol to 24HC, which in turn triggered the production of TNFα. Additionally, our results elucidated a critical role of FAK and NFκB in mediating 24HC-dependent proinflammatory response. In summary, our studies have identified 24HC as another oxysterol that regulates proinflammatory response in macrophages via the integrin-FAK-NFκB signaling pathway.

## Materials and methods

### Construction of full-length integrin αvβ3 model

*Modeling of the missing residues in the Extracellular Domain (ECD) of integrin αVβ3.* Borst et al.^62^ reported the electron microscope structure of human integrin αVβ3 in three distinct conformational states: bent, extended, and open, along with therapeutic antibody LM609. The integrin αvβ3 bent state (PDB ID 6AVQ) structure was used for the molecular docking of 24HC in site II, followed by atomistic molecular dynamics simulation. Before performing MD simulations, missing residues in the αv subunit (residues 839–867, 957) and in the β3 subunit (residues 689–692) were modeled using modeler^63^. Out of 100 modeled residues, the best structure was selected as the 99th PDB file with a DOPE score of − 165,357.

### Transmembrane (TM) helices modeling

The transmembrane helices of integrin subunits are short α-helix spanning the entire lipid bilayer and are arranged in a right-handed coiled-coil conformation. The inactive state of the integrin is characterized by the association of these TM helices in the membrane^64^. The NMR structure of αIIbβ3 integrin’s TMD revealed that the membrane-proximal regions of the β3 subunit are tilted by ~ 25° with respect to the bilayer normal^65,66^. However, the αIIb subunit is oriented in parallel to the bilayer normal. The GXXXG-like motif makes the outer membrane clasp (OMC). The inner membrane clasp (IMC) has a highly conserved GFFKR motif with the two Phe residues found in all α subunits^67^ (Fig. S1). Any alteration in the OMC leads to incorrect TM helix association. MD simulation studies demonstrated that the different compositions of the GXXXG motif in α subunits contribute to the differential integrin activation^68^. Similarly, studies have shown the importance of the IMC, with two conserved Phe residues in the GFFKR motif maintaining the correct resting state of TMD^69^. Since the TMD structure of integrin αVβ3 is not available, we built its homology model using the NMR structure of αIIbβ3 as a template^70^ (PDB ID 2K9J), which has a sequence identity of 54%. The OMC and IMC motifs as AVLAG and GFFKR, respectively, are almost similar to αIIbβ3. The OMC motif helps in the correct association of TM helices, and IMC helps in differential integrin activation with ligand binding specificity. The membrane-located region, as determined by the OPM web server, is highlighted with an upper and lower leaflet of the membrane. A strong salt bridge interaction with residues pair αv_R995 (guanidyl group) and β3_D273 (carboxyl group) on the IMC results in the resting state of integrin αvβ3. Interestingly, the same salt bridge interaction between residue pair αIIb_R995 and β3_D273 in the IMC immediate vicinity along the membrane-proximal region has been shown to have functional importance resulting in the resting conformational state of integrin αIIbβ3^71^. In addition to the αv_R995 (guanidyl group) and β3_D273 (carboxyl group) salt bridge, MD studies revealed the other three salt bridges responsible for TM helix association. However, these studies do not comment on the role of lipids and intracellular proteins Talin1 and FAK that alters these salt bridge interactions.

### Molecular docking of 24HC to Integrins

The binding poses and molecular interactions of 24HC in the integrin αvβ3 and α5β1 site II were determined by docking using MOE^43^ software. The 24HC docking was performed on several snapshots of the receptor collected from the membrane-equilibrated integrin model. The protein structure was prepared using MOE *QuickPrep*. The protonation states were assigned using *Protonate3D.* The 24HC binding sites were specified using residues S162, A263, V161, I265, T285, T286, Q267 from the β3 subunit and L111, W93, K42, Y18, F427, M400, S399 from the αv subunit. The London dG scoring function was used for the initial placement (triangle match method) of the ligands within the binding sites. The docked molecule was further refined by induced fit docking and ranked using the Generalized Born Volume Integral/Weighted Surface Area, a forcefield-based scoring function^72^.

### MD simulations protocols

#### Unbiased MD simulations

All-atom MD simulations were performed with GROMACS v5.1.2^73^ version built on GPU-supported workstations. The pre-oriented protein coordinates relating to the membrane normal were obtained from the OPM database^44^. The CHARMM-GUI membrane builder^74^ was used to build simulation systems on these oriented structures. It generates multiple input files for performing stepwise minimization, equilibration, and production run. The mixed lipid bilayer composition containing POPC and 20% cholesterol (CHOL) was used. The lower and upper leaflets contained 284 (190 POPC + 94 CHOL) and 280 (188 POPC + 92 CHOL) lipids. The extracellular and intracellular sides of the bilayer were solvated with 40 Å water padding. The salt concentration of 0.15 M NaCl was maintained with Na^+^ and Cl^-^ counter ions. The CHARMM36 force field^75^ and TIP3P^76^ water models were used. The multistage (8 steps) simulation protocol starts with the 5000 steps of energy minimization. The six steps of equilibration proceed with two steps of NVT (1 fs time step) and four steps of NPT (first run with 1 fs and remaining with 2 fs time step) simulations. During equilibration, position restraints were applied to the membrane, and protein atoms were gradually removed to ensure proper system equilibration. During the equilibration phase, the Berendsen thermostat^77^ and barostat was used to maintain the 310 K temperature and 1 bar pressure. Finally, the production run was performed. A temperature of 310 K was maintained with the Nose–Hoover thermostat^78^ at 1 ps of the coupling time constant. A pressure of 1 bar was kept with the Parrinello-Rahman barostat^79^ using semi-isotropic scaling and coupling time constant of 5 ps. Long-range electrostatic interactions were calculated using the Particle Mesh Ewald method^80^ with a cutoff of 12 Å. All the covalent bonds involving hydrogen atoms were constrained with the LINCS algorithm^81^ to integrate at a 2-fs time step. For production runs, frames were saved at every 20 ps. The parameters for ligands were obtained from CHARMM general force field (CGenFF) ParamChem server (https://cgenff.paramchem.org)^82^.

### Ligand access and binding mode predictions by well-tempered metadynamics

As previously published^60,61^, we performed association simulations using well-tempered metadynamics (WT-metaD) to assess the potential binding orientations of 24HC and 25HC at the integrin allosteric site (site II). Briefly, the starting configurations of the system were obtained by placing 24HC or 25HC randomly in the aqueous phase approximately 30–40 Å away from site II of the unbound receptor species. The system was minimized and equilibrated, and the equilibrated configuration was used as input for WT-metaD simulations. We used two collective variables (CVs): (1) the distance between the center-of-mass (COM) of the ligands and COM of the active site residues, and (2) the orientational angle of the ligands measured as the angle formed by the backbone carbon atom of I265, 3’OH, and C23 (for 24HC) or C25 (for 25HC) atoms. The distance between the COM of the ligand and the backbone carbonyl (C=O) oxygen of the binding site residue G264 was used as a committer with a cutoff distance of 4 Å. The bias applied to the system was turned off, and the simulation was terminated once the minimum cutoff distance was reached. Simulations were performed at 310 K, with the lower and upper wall limits of 3.0 and 3.5 nm, a sigma value of 0.5, a height of 1.5, and two different bias factors, 10 and 15. All simulations were performed using GROMACS v2021 coupled with PLUMED v2.7.2. For every combination of committer and bias factors, simulation was performed in triplicates.

### Trajectory analysis

#### Contact frequency

The contact frequency of 24HC with the integrin αVβ3 was calculated using the in-house Tcl script. The cutoff distance used was 4 Å. The radar plot figure was created using PlotLy^83^.

*Secondary structure content.* The secondary structure content of the SDL loop (resid 158 to 190) in the αVβ3-24HC complex was calculated using the VMD STRIDE program and selecting CA atoms of the SDL loop.

### Surface plasmon resonance (SPR) studies

Real-time biomolecular interactions between integrin αvβ3 and 24HC or 25HC were investigated by SPR using a Biacore S200 instrument (Cytiva). To create an appropriate interaction surface (R_max_ ~ 25), purified human αvβ3 Integrin (purchased from Yo proteins AB, Huddinge, Sweden) was covalently immobilized on a flow cell of CM5 chip (carboxy-methylated dextran coated) in 10 mM sodium acetate buffer, pH 4.0, using EDC/NHS amine coupling chemistry at 25 °C. The unused dextran surface was then inactivated by injecting 1 M ethanolamine, pH 8.5. The corresponding blank control flow cell was then activated and inactivated without the protein for background binding correction. For kinetic analysis, increasing concentrations of 24HC (or 25HC), as indicated in the figures (Fig. 6), in the running buffer PBS-P + (20 mM phosphate, 2.7 mM KCl, 137 mM NaCl, 0.05% polysorbate 20, pH 7.4) with 1% DMSO were injected at a flow rate of 30 μL/min for 180 s. Following dissociation for 600 s, the chip surface was regenerated with the running buffer. The SPR sensorgrams were plotted and quantitatively evaluated to determine the affinity constant (*K*_D_) using the Biacore S200 Evaluation Software (Cytiva) and the steady-state equilibrium binding model.

### Materials and methods for studies with macrophages

*Cells.* RAW 264.7 macrophages (ATCC, Virginia, USA) were maintained in complete DMEM containing 10% FBS, 100 IU/ml Penicillin, and 100 ug/ml Streptomycin (Gibco).

*Cell treatment.* RAW 264.7 macrophages were treated with either 24HC (50 µM) (Steraloids, Rhode Island, USA) or mouse TNF-α (100 ng/ml) (R & D systems). In some experiments, cells were pre-treated with either FAK inhibitor (5 µM) (PF-431396; Sigma Aldrich) or NFkB inhibitor (1 µM) (Bay11-7082; InvivoGen) for 1 h. Subsequently, these cells were treated with 24HC.

### Cytokine detection assay

TNF levels in the medium supernatant of RAW 267.3 macrophages were assessed by using a specific ELISA kit (eBioscience, California, USA). ELISA data were analyzed using GraphPad Prism software (6.0), and a significance test was carried out using Student’s t-test.

### Reverse transcription- PCR (RT-PCR)

Total RNA was extracted using TRIzol reagent (Life Technologies, California, USA) following the manufacturer’s instructions. MultiScribe reverse transcriptase (Applied Biosystem, California, USA) was used to synthesize template cDNA. PCR was performed using Apex® 2X Taq Red master mix (Genesee Scientific, California, USA) in a final reaction volume of 25 ml. Following amplification, PCR products were analyzed on 1.2% agarose gel and bands in the gel were visualized by ChemiDoc XRS (Bio-Rad, California, USA). Amplified glyceraldehyde-3-phosphate dehydrogenase (GAPDH) gene PCR product was used as a loading control. The primers used to detect the indicated genes are listed below:

Mouse GAPDH forward; 5′-GCCAAGGTCATCCATGACAACTTTGG, Mouse GAPDH reverse, 5′-GCCTGCTTCACCACCTTCTTGATGTC.

Mouse CYP46A1 forward; 5′-TGTCATCGCTGGCTTTTCAG, Mouse CYP46A1 reverse, 5′- GACGATGGTAGTTGTGGTGATAGC.

## Supplementary Information

Supplementary Information.

## Data Availability

The data generated during the current study are available from the corresponding author upon reasonable request.
