# Supplementary material for: Molecular basis for the recognition of 24-(S)-hydroxycholesterol by integrin αvβ3
Source: Sci Rep. 2023 Jun 6;13:9166. doi: 10.1038/s41598-023-36040-4 (PMC10244445; doi:10.1038/s41598-023-36040-4)
Supplement: Supplementary file 1 — Supplementary Information. [file 41598_2023_36040_MOESM1_ESM.docx]

**Supplementary Information:**

**Molecular Basis for the Recognition of 24-(*S*)-Hydroxycholesterol by Integrin αvβ3**

Jeevan B. GC^1,ϕ^, Justin Chen^1,ϕ^, Swechha M. Pokharel^2^, Indira Mohanty^2^, Charles Mariasoosai^1^, Peter Obi^1^, Paul Panipinto^1^, Smarajit Bandyopadhyay^3^, Santanu Bose^2^, and Senthil Natesan^1,*^

*^1^Department of Pharmaceutical Sciences, College of Pharmacy and Pharmaceutical Sciences, Washington State University, Spokane, WA 992020, USA.*

*^2^ Department of Veterinary Microbiology and Pathology, Washington State University, Pullman, WA 99210, USA.*

*^3^Molecular Biotechnology Core Laboratory, Lerner Research Institute, Cleveland Clinic, Cleveland, OH 44195, USA.*

**Table S1.** Physicochemical properties of 24(S)-hydroxycholesterol and 25-hydroxycholesterol

| **Property** | **24(S)-hydroxycholesterol** | **25-hydroxycholesterol** |
| --- | --- | --- |
| molecular formula | C_27_H_46_O_2_  cholest-5-ene-3beta,24-diol | C_27_H_46_O_2_  Cholest-5-ene-3beta,25-diol |
| molecular weight (g/mol) | 402.7 | 402.7 |
| XlogP | 7 | 6.8 |
| HBD | 2 | 2 |
| HBA | 2 | 2 |
| Rotatable bond count | 5 | 5 |
| topological polar surface area (Å^2^) | 40.5 | 40.5 |

**Table S2.** Lipid composition of the model membrane bilayer used in the simulations.

| **Lipids** | **Upper Leaflet** | **Lower Leaflet** |
| --- | --- | --- |
| Cholesterol | 92 | 94 |
| POPC | 188 | 190 |
| Total | 280 | 284 |

**Table S3.** Simulation Details

| Box Size | 131 X 131 X 211 Å^3^ |
| --- | --- |
| Salt Concentration | 0.15M NaCl |
| No. of atoms | 340646 |
| No. of water | 83123 |
| No. of Ions | 504 |

.
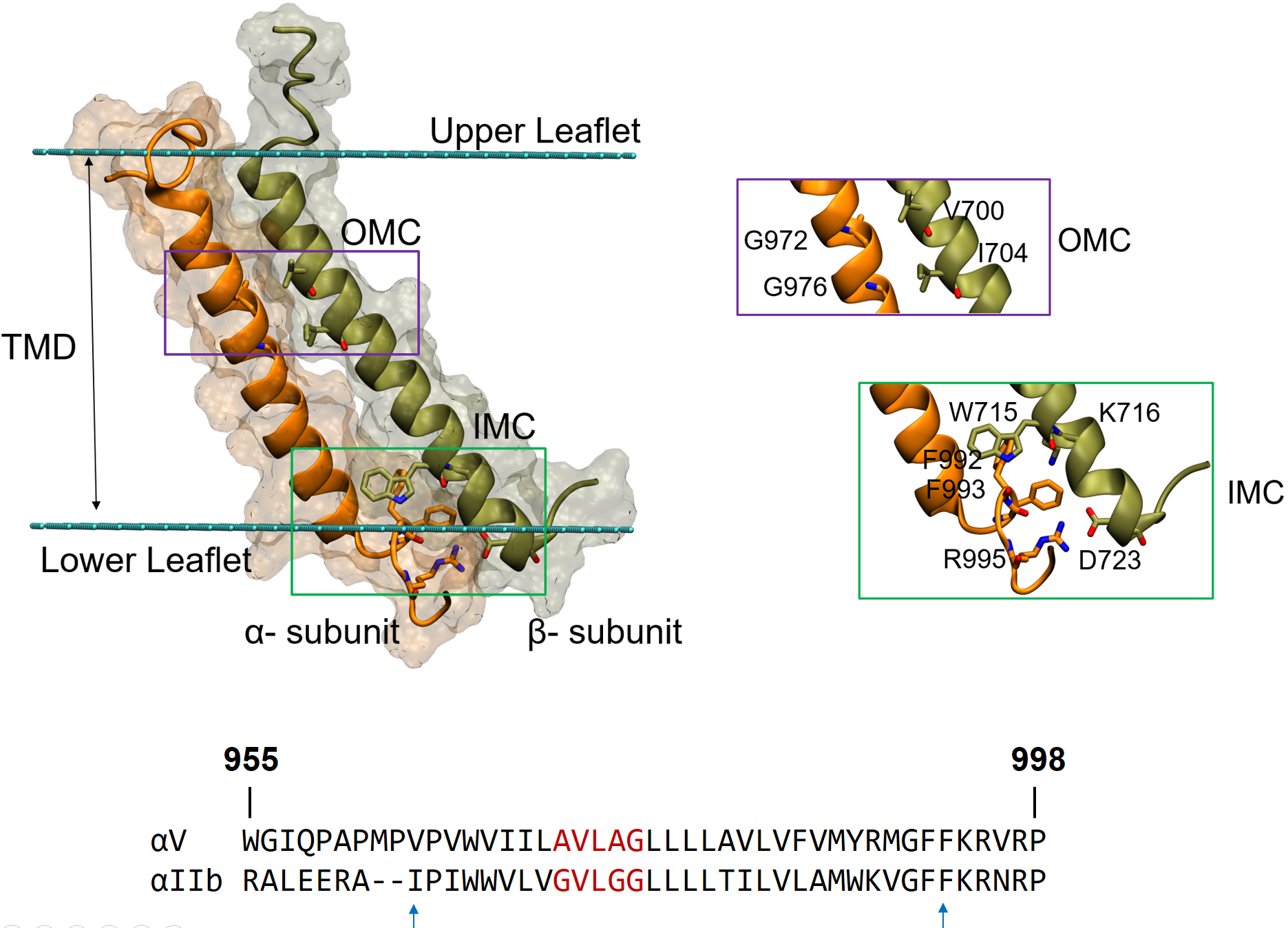


**Fig. S1**. **Homology model of transmembrane helices of integrin αvβ3 built using the NMR structure of αIIbβ3 as a template** ^33^ (PDB ID 2K9J). The outer membrane clasp (OMC) consists of GXXXG-like motif helps in correct transmembrane helix association. The inner membrane clasp (IMC) consists of GFFKR motif with two conserved Phe residues from the α-subunit, critical for maintaining the correct resting state of the transmembrane helices.


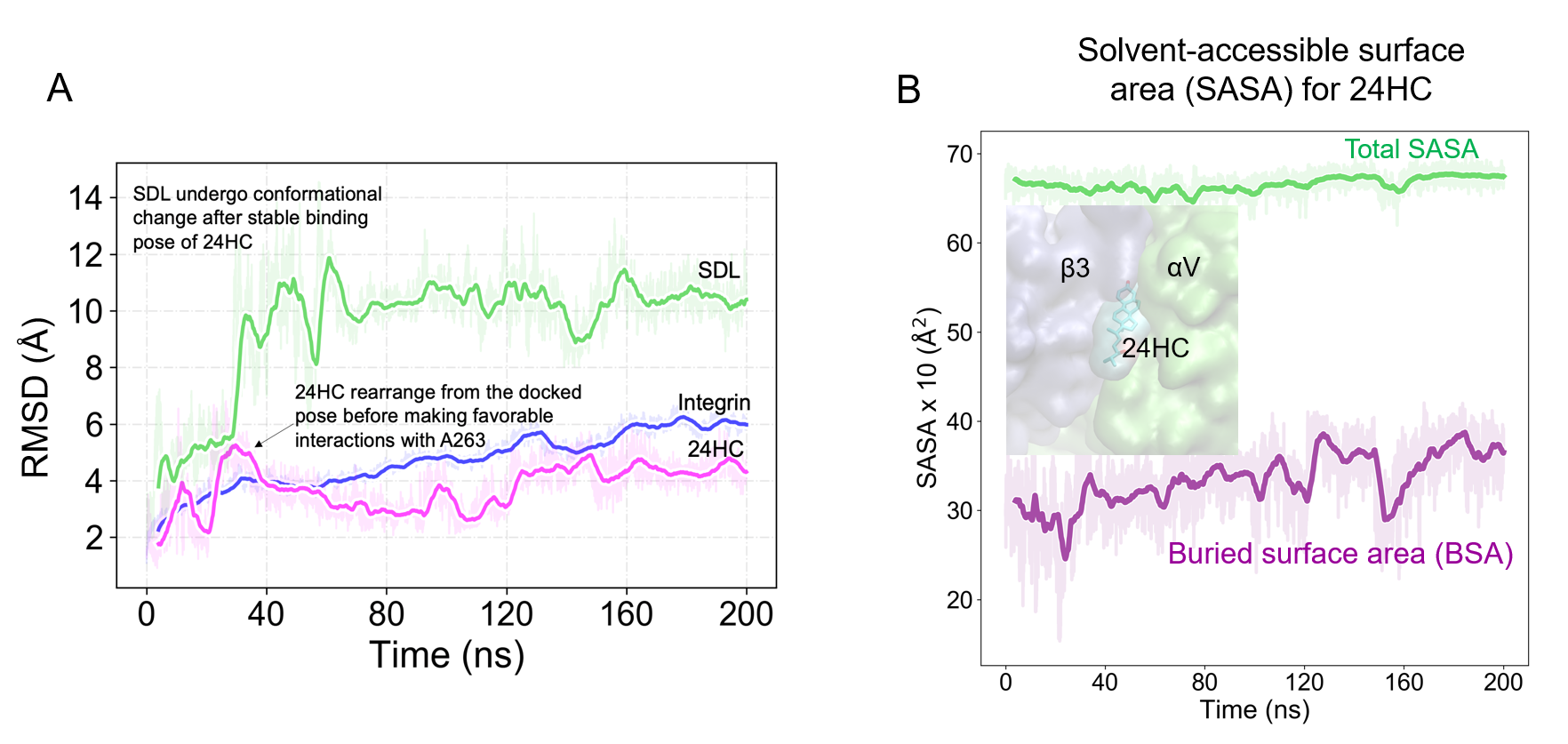


**Fig. S2.** **Stability of the integrin αvβ3 and 24HC complex**. A) The RMSD of integrin was calculated using the backbone and sidechain protein heavy atoms, removing all translational motion of the protein. The initial docked complex as a reference frame. B) COM distance between the integrin binding site-II and 24HC. C) Solvent accessible surface area (SASA) and buried surface area (BSA) of 24HC. More than 50% of the accessible surface area of 24HC is buried inside the integrin binding site-II.

**
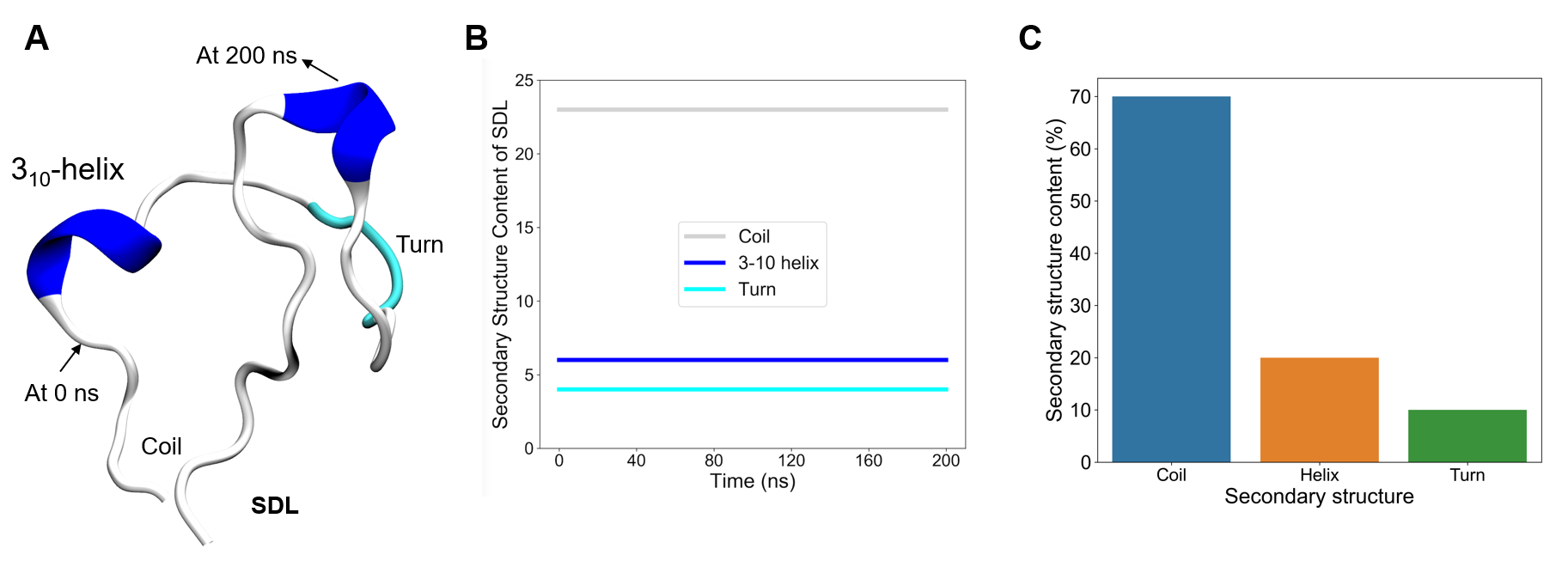
**

**Fig. S3. The secondary structure content of the SDL loop in the integrin αVβ3-24HC complex.** A) The C-alpha (CA) atoms of the SDL loop (residues 158 to 190) were selected to calculate the secondary structure content using the VMD STRIDE program. B) All the secondary structure contents remain unchanged during the 200 ns MD simulations. C) The majority of SDL is coil (up to 70%), followed by 20% 3-10 helix and ~10% of turn.

**
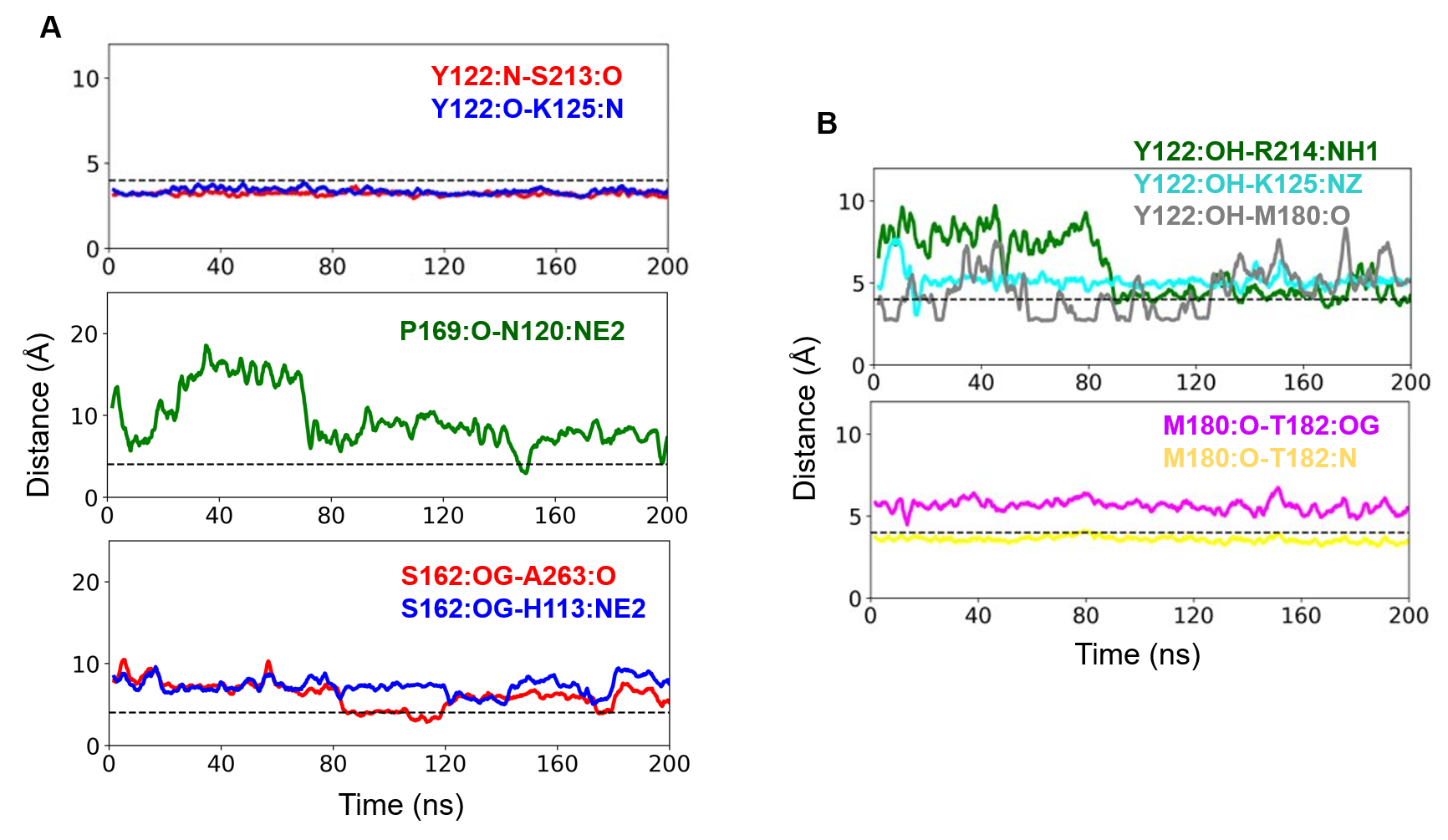
**

**Fig. S4. The interactions between the SDL loop residues Y122, S162, P169, M180, and T182 and various residues of site II and β-propeller.** A) The H-bond interactions between the backbone amino group and carbonyl groups of Y122 with the backbone carbonyl group of S213 and the amino group of K125 in red and blue, respectively. The distance between the backbone carbonyl oxygen of P169 of SDL and the side chain amino group of N120 of β-propeller (green) varies as SDL undergoes significant conformational changes. The distances between the sidechain -OH group of S162 of site II with the backbone carbonyl oxygen of A263 (site II residue) and the sidechain amino group of H113 of β-propeller are given in red and blue, respectively. B) The distances between the sidechain -OH group of Y122 and the sidechain amino groups of R214, K125, and the backbone carbonyl group of M180, are given in green, cyan, and gray, respectively. The distances between the backbone carbonyl oxygen of M180 and the sidechain -OH group and backbone amino group of T182 are given in magenta, and yellow, respectively.

**
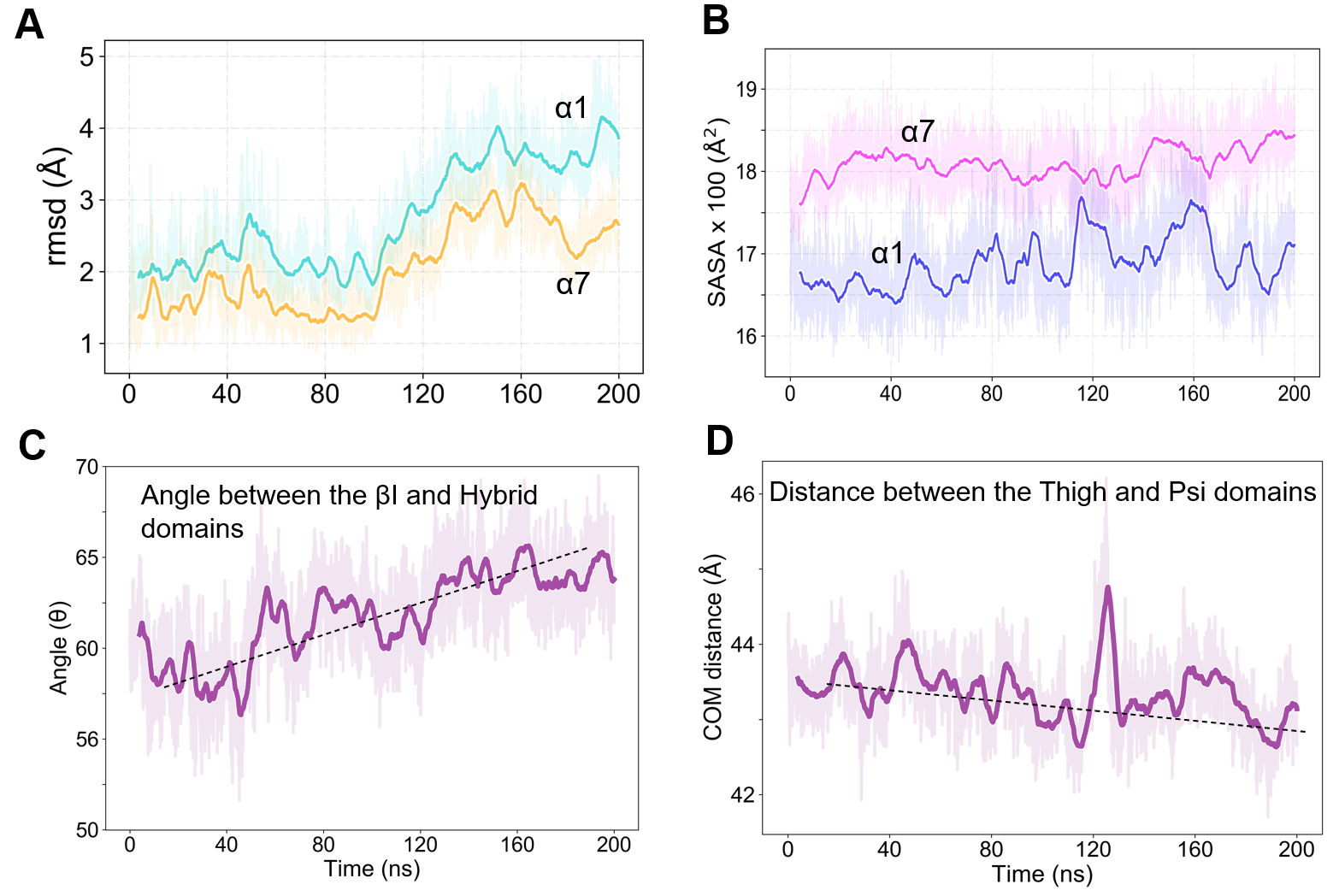
**

**Fig. S5.** Various activation signatures in the integrin αVβ3 -24HC complex during the 200 ns MD simulation. **A)** Both α1- and α7-helices undergo moderate conformational changes along the trajectory, showing up to 4 Å RMSD changes. B) However, no significant changes are observed in the solvent-accessible surface area (SASA) of the helices. C) The angle between the βI and Hybrid domains increased from ~60 to 65° in an increasing trend. D) The center-of-mass (COM) distance between the Thigh and Psi domains remained between 43-44 Å, indicating no significant changes. It should be noted that the simulation time of 200 ns may not be sufficiently long to observe any significant conformational changes in this large protein-ligand complex.


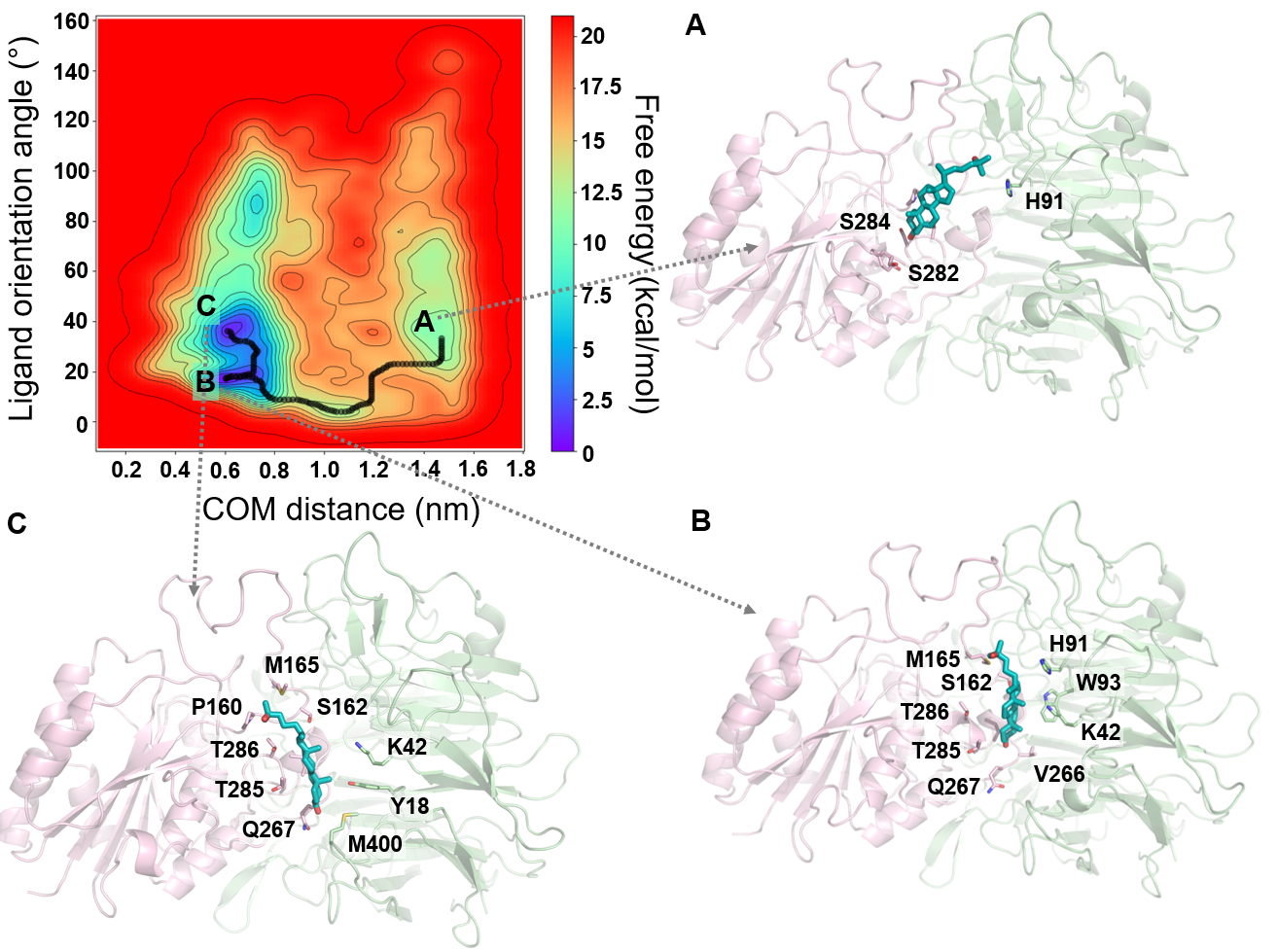


**Fig. S6**. **The free energy surface (FES) for 25HC’s access and binding to the integrin αVβ3 site II**. The FES was characterized by the distance between the center-of-mass (COM) of the ligand and COM of the binding site residues (X-axis), and the orientational angle of 25HC in degrees formed by the backbone carbon atom of I265, 3’OH and C25 of 25HC (Y-axis, in degrees). The minimum energy path for the access of 25HC to the binding site was determined by well-tempered meta dynamics simulations, shown in black dots within in the 2D energy surface. The final bound and the intermediate states were labelled A-C. A) Initially, as 25HC approached site II by making contacts with residues H91, S282 and S284. B) 25HC explored the binding site mostly in an orientation where 3-OH of 25HC was positioned toward the lower end of the binding site. C) 25HC further negotiated its contacts with the binding residues and assumed the final pose that was similar to the most favorable binding orientation reported in our previous study. C) In the final bound conformation, 25HC makes electrostatic and hydrophobic contacts with the active site residues Y18, K42, W93, and M400 of β-propeller domain and P160, S162, M165, Q267, T285 and T286 of βI domain.


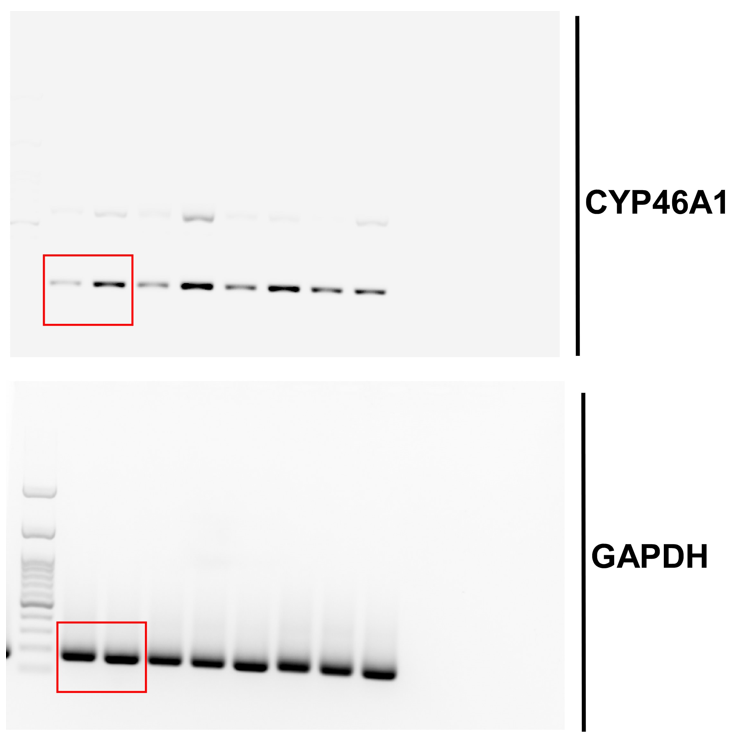


**Fig. S7. The original PCR gels related to Figure 7 in the main text are shown above.** The portions of the gel shown in Figure 7 are indicated above with red boxes.
